# *SAXS-A-FOLD*: a website for fast ensemble modeling optimizing the fit of *AlphaFold* or user-supplied protein structures with flexible regions to SAXS data

**DOI:** 10.1107/S1600576725003590

**Published:** 2025-05-29

**Authors:** Emre Brookes, Joseph E. Curtis, Aaron Householder, Mattia Rocco

**Affiliations:** a Department of Chemistry and Biochemistry University of Montana 32 Campus Drive Missoula Montana59812 USA; b NIST Center for Neutron Research National Institute of Standards and Technology Gaithersburg Maryland20878 USA; c Department of Computer Science University of Central Florida 4000 Central Florida Blvd Orlando Florida32816 USA; d Proteomica e Spettrometria di Massa IRCCS Ospedale Policlinico San Martino Largo R. Benzi 10 Genova16132 Italy; NSRRC, Taiwan

**Keywords:** small-angle X-ray scattering, SAXS, *AlphaFold*, ensemble modeling, protein structures, structural flexibility

## Abstract

AI programs such as *AlphaFold* (*AF*) are having a major impact on structural biology. However, predicted unstructured regions, the arrangement of linker-connected domains and their conformational changes in response to environmental variables present challenges that are not easily dealt with on purely computational grounds. An approach that uses predicted (or solved) protein modules/domains linked by potentially unstructured regions and that generates ensembles of models optimized against small-angle X-ray scattering (SAXS) data has been recently described [Brookes *et al.* (2023). *J. Appl. Cryst.***56**, 910–926]. Its implementation on a public-domain website, *SAXS-A-FOLD* (https://saxsafold.genapp.rocks), is presented here. User-supplied SAXS experimental intensity *I*(*q*) versus scattering vector magnitude *q* and the derived pair-wise distance distribution function *P*(*r*) versus *r* are first uploaded. An *AF* or user-supplied structure (currently only single chains without prosthetic groups) is then uploaded and displayed, and its SAXS *I*(*q*) and *P*(*r*) profiles are computed and compared with the experimental data. If uploaded from *AF*, the structure is color-coded by the associated confidence level: on this basis, the website automatically proposes potential flexible regions that can be user modified. For user-supplied structures, these regions have to be directly entered. A starting pool of typically 10–50 × 10^3^ conformations is generated using a Monte Carlo method that samples backbone dihedral angles along the chosen segments of potential flexibility in the protein structures. The initial pool is reduced to obtain a tractable set of models, for which *P*(*r*) and *I*(*q*) are computed with fast established methods. A global fit is performed using non-negatively constrained least-squares (NNLS) versus original data. The *P*(*r*) and *I*(*q*) NNLS results are then displayed, showing both the reconstructed curves and the contributing model curves, with their percentage contributions. A *WAXSiS* (https://waxsis.uni-saarland.de) implementation is utilized to calculate an *I*(*q*) for each selected model. These sets can be enhanced by adding a user-defined number of models generated before and after each selected model in the original Monte Carlo pool, ensuring the inclusion of nearby models that might better fit the data. Finally, NNLS is used on the *WAXSiS*-generated *I*(*q*) set versus the original *I*(*q*) data, with the results displaying the contributing models and their *I*(*q*). Aside from being representative of contributing conformations, the models selected by *SAXS-A-FOLD* could constitute a set of starting structures for more advanced MD simulations.

## Abstract 2

Predicted protein structures containing flexible regions, or solved structural units linked by disordered segments, will not have a unique conformation in solution. Starting from a single structure, the *SAXS-A-FOLD* webserver can generate thousands of conformations using a Monte Carlo method. It can compare their calculated SAXS profiles against experimental data, resulting in an ensemble of representative models.

## 1. Introduction

Neural-network-based artificial intelligence (AI) programs such as *AlphaFold* (*AF*) (Jumper *et al.*, 2021 ▸) and *RosettaFold* (Baek *et al.*, 2021 ▸) have revolutionized the protein structure field, culminating in the 2024 Nobel Prize in chemistry (see Callaway, 2024 ▸). In particular, the *AF* consortium has created a repository with freely available structural predictions over the available protein sequence universe (*AF2*; https://alphafold.ebi.ac.uk). Given the overall accuracy attained by the predicted structures, this has already generated many applications from templates in traditional experimental determination such as X-ray crystallography, NMR and cryo-electron microscopy (see Corum *et al.*, 2024 ▸; Kovalevskiy *et al.*, 2024 ▸; Laurents, 2022 ▸; Terwilliger *et al.*, 2022 ▸) to the direct inference of structure–function relationships (see *e.g.* Yang *et al.*, 2023 ▸). While initially the predictions were produced only for single-chain proteins, current efforts with *AlphaFold3* are tackling multi-chain structures and complexes (Abramson *et al.*, 2024 ▸; https://alphafoldserver.com). Some relatively minor drawbacks are the inclusion of signal peptide and/or pre-protein sequences while the mature protein form usually lacks them, and the absence of other post-translational modifications. For the former, using the *AF2* structure subset for which curated information was present in the UniProt database (https://www.uniprot.org), some of us have created a repository containing the mature structures and their computed hydro­dynamic properties, circular dichroism spectra and small-angle X-ray scattering (SAXS) pair-wise distance distribution function *P*(*r*) versus *r* [hereafter *P*(*r*)] (Brookes & Rocco, 2022 ▸; https://somo.genapp.rocks/somoaf). For the latter, much community work is still needed, but very recently an on-line tool dealing with glycosyl­ation, which can significantly affect the overall size and shape of a protein, has been made available (Ives *et al.*, 2024 ▸; https://glycoshape.org).

However, another relevant issue is often present: that of the predicted unstructured regions, which can either behave as freely moving appendages or act as flexible linkers between fully structured domains or modules. Both are widely present in the protein universe, and often the linkers can act as regulatory elements affecting the overall conformation in response to binding by other small or large structural entities. In these cases, a static representation fails to capture the protein’s full structural complexity, which can be explored experimentally at the atomic level by NMR (but with relevant size limitations). Cystallography and cryo-EM can usually at best offer ‘snapshots’ of particular energy-favored conformational states, although there is progress also in this respect (*e.g.* Punjani & Fleet, 2023 ▸). All-atom molecular dynamics (MD) simulation methods can, in principle, produce a more advanced description of the structural landscape explored (see Schlick *et al.*, 2021 ▸), but they are very computationally demanding and appear to favor more compact conformations (see *e.g.* Zhang *et al.*, 2017 ▸).

In a recent paper, some of us have described an approach that uses predicted (or solved) protein modules/domains linked by potentially unstructured regions and that generates ensembles of models optimized against SAXS data (Brookes *et al.*, 2023 ▸; see also Receveur-Bréchot, 2023 ▸). The Brookes *et al.* (2023 ▸) study used three *AF2* structures for which corresponding SAXS data were available in the Small-Angle Scattering Biological Data Bank (SASBDB) (Valentini *et al.*, 2015 ▸; https://www.sasbdb.org) that did not initially match those calculated from the structures, with respect to both the *P*(*r*) and the intensity *I* versus scattering vector magnitude *q* [hereafter *I*(*q*), where *q* = (4π sin θ)/λ with θ being half the scattering angle and λ the wavelength of the incident radiation]. The confidence level indicators that *AF2* provided with the predicted structures were used to identify potential flexible linkers between structured domains. The *Monomer Monte Carlo* (*MMC*) program developed at NIST (Curtis *et al.*, 2012 ▸; Perkins *et al.*, 2016 ▸; https://sassie-web.chem.utk.edu/sassie2), which uses the CHARMM force field (https://academiccharmm.org) to create a Ramachandran-like sampling of the dihedral angles φ and ψ along chosen segments of the protein backbone, was employed. As a result, 20000 models of varying conformation from each starting structure were generated, of which on average ∼15000 were accepted after screening for steric clashes. From each *MMC*-generated starting pool of models, a sub-selection stride (*i.e.* selecting every *n*th model) was applied to produce a reduced pool of ∼1000–1700 models, for which *P*(*r*) and *I*(*q*) were computed using fast methods. A non-negatively constrained least-squares (NNLS) procedure (see Brookes *et al.*, 2016 ▸; Brookes *et al.*, 2023 ▸; Lawson & Hanson, 1995 ▸) was then used to filter the reduced pool of models to those for which the sum of their calculated *P*(*r*) and *I*(*q*), weighted by their percent contribution, best matched the respective experimentally derived data, producing a set of ‘preselected models’. As a final step, each preselected model was further evaluated by calculating its *I*(*q*) with the computationally intensive *WAXSiS* program, which uses a short explicit solvent MD simulation (Chen & Hub, 2014 ▸; Knight & Hub, 2015 ▸; https://waxsis.uni-saarland.de). NNLS was then again applied to the *WAXSiSI*(*q*) calculations of the preselected models, identifying a final weighted set of models that predicted the experimental SAXS data up to an order of magnitude better than those derived from the starting structures (Brookes *et al.*, 2023 ▸).

Good to excellent quality SAXS data can be obtained on stoichiometrically monodisperse protein structures (*i.e.* separated from different complex states, *e.g.* monomers from dimers or higher oligomerization states) at several synchrotron facilities worldwide, often in the preferred mode of being directly coupled to on-line size-exclusion chromatography (Mohammed *et al.*, 2024 ▸; Pérez & Vachette, 2017 ▸), and pipelines including mailing-in of samples are becoming increasingly available (*e.g.*https://www.diamond.ac.uk/Instruments/Soft-Condensed-Matter/small-angle/B21.html; https://www.embl-hamburg.de/biosaxs/mailin.html; https://www.synchrotron-soleil.fr/en/beamlines/swing; https://htsaxs.bl1231.als.lbl.gov/). SAXS experiments report on the time and ensemble average of the structures present in solution, and therefore could be employed to test predictions about the conformational states present (see Koch *et al.*, 2003 ▸). It is thus conceivable that coupling structure prediction methods, or individually solved modules, with meso-resolution experimental data with relatively fast acquisition times could become a standard pipeline to gain a deeper insight into the landscape of proteins containing unstructured and/or flexible regions. In this direction, we present the public-domain *SAXS-A-FOLD* website (https://saxsafold.genapp.rocks), where the procedures described by Brookes *et al.* (2023 ▸) are implemented and streamlined, allowing the generation of representative structural models within a unified computational framework. Currently, only single-chain proteins without prosthetic groups can be processed, but an expansion toward dealing with complexes and post-translational modified structures is under consideration.

## 2. Methods

**Figure 1:**
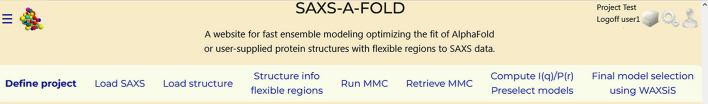
General layout of the *SAXS-A-FOLD* website, with the Tabs giving access to the operational sections.

**Figure 2:**
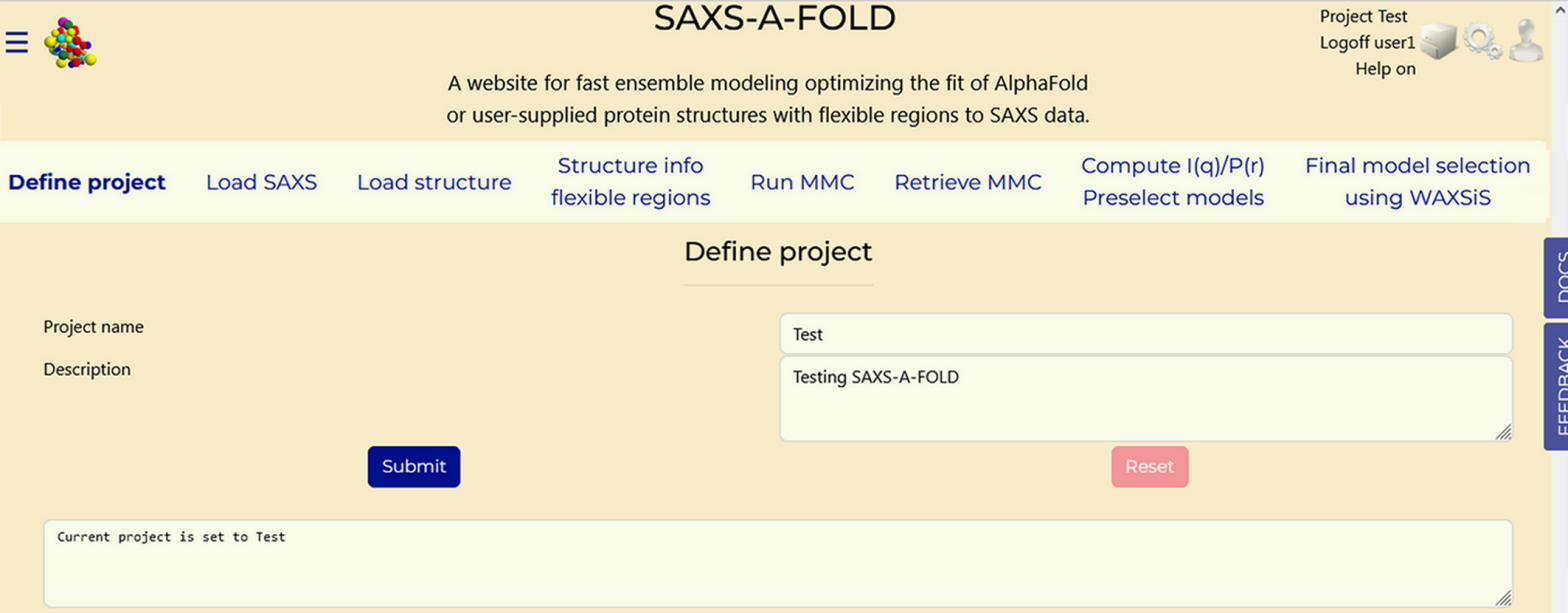
Defining a project in the first Tab.

The website was built using the *GenApp* framework (Savelyev & Brookes, 2019 ▸) and is hosted on Indiana University’s Jetstream2 cloud (Hancock *et al.*, 2021 ▸) made available through an award from the National Science Foundation’s ACCESS program (Boerner *et al.*, 2023 ▸). The virtual machine uses, at the time of writing, 32 AMD EPYC-Milan cores and 128 GB of RAM; however, this may be adjusted or moved to an elastic model (Brookes & Savelyev, 2017 ▸) depending on community usage. The software stack is running in a Docker (Merkel, 2014 ▸) container built originally from an Ubuntu 20.04.4 LTS image. The *GenApp* framework builds web applications from definition files running command line executables (henceforth, executables). *GenApp* directly supports *PlotlyJS* (version 2.35.2; https://plot.ly) for plots and *JSmol* (version 14.0.2; Hanson *et al.*, 2013 ▸) for molecular visualization. *GenApp*-generated websites have features available for management of a ‘cloud’ file system, job management, user management, feedback reporting and integrated documentation. These are available, respectively, via the ‘file’, ‘gear’ and ‘head’ icons at the top right (see Fig. 1), and the ‘FEEDBACK’ and ‘DOCS’ tabs on the right of the webpage (see Fig. 2). *GenApp* also has an option to provide an e-mail notification on completion of a job. This feature is active for the potentially time-consuming ‘Load structure’ (due to the *WAXSiS* computation included), ‘Run MMC’, ‘Retrieve MMC’, ‘Compute I(q)/P(r), Preselect models’ and ‘Final model selection using WAXSiS’ modules. The *SAXS-A-FOLD* code is freely available in a public GitHub repository (https://github.com/ehb54/saxsafold). Each ‘module’ of the website has a defining *JSON* (Pezoa *et al.*, 2016 ▸) formatted module file which references an executable. All modules’ executables are written in PHP 7.4.3 (Lerdorf & Tatroe, 2002 ▸) with the exception of the ‘Run MMC’ module, which is written in Python 2.7.18 (Van Rossum & Drake, 1995 ▸). The ‘Load structure’ executable leverages work done previously (Brookes & Rocco, 2022 ▸; Brookes *et al.*, 2023 ▸) and utilizes multiple software packages described therein, including *US-SOMO* (Brookes & Rocco, 2018 ▸), for calculation of the molecular mass, partial specific volume, theoretical hydration, radius of gyration and *P*(*r*) from the structure; *Chimera* (version 1.16; Pettersen *et al.*, 2004 ▸) for identification of α-helices and β-sheets; and* MAXIT* (version 11.100) for interconversion of PDB-, CIF- and mmCIF-formatted structure files. ‘Retrieve MMC’ utilizes *mdconvert*, included in the *MDTraj* (version 1.10.0; McGibbon *et al.*, 2015 ▸) suite, to convert DCD-formatted frames output by ‘Run MMC’ to PDB files consumable by *US-SOMO* for *P*(*r*) computations and by *JSmol* for visualization. Notably, the routines for running various *I*(*q*) calculators have been written to enable the future inclusion of additional calculation programs with minimal effort.

A new PHP class named *SAS* (https://github.com/ehb54/saxsafold/blob/main/bin/sas.php) was created for this project which provides functions for managing *I*(*q*) and *P*(*r*) data as well as *Plotly* plot generation. This class is used extensively by the PHP executables and proved invaluable for testing, validation and ease of use during module development. The class *SAS* additionally provides functions which call *US-SOMO*’s command line executable interface for *P*(*r*) and NNLS computations.

SAXS-compatible *P*(*r*) functions are calculated on dry structures as described by Brookes *et al.* (2023 ▸), utilizing code developed for *US-SOMO*. Currently, the default (fixed) options are a 1 Å bin size and normalizing the resulting *P*(*r*) versus *r* profile by the calculated molecular weight of the structure analyzed. For fast *I*(*q*) calculations, we offer the choice between *PEPSI-SAXS* (version 3.0; Grudinin *et al.*, 2017 ▸) and, for academic users only, *CRYSOL* (Svergun *et al.*, 1995 ▸) version 3.2.1 (Manalastas-Cantos *et al.*, 2021 ▸). For *CRYSOL*, the server currently employs 25 for the maximum number of spherical harmonics, 18 for the order of Fibonacci’s grid and 0.02 e Å^−3^ for the hydration shell contrast. For *PEPSI-SAXS*, default values are used, including 5% of the bulk value for hydration shell contrast and a dynamic determination of the expansion order. The *WAXSiS* (Chen & Hub, 2014 ▸; Knight & Hub, 2015 ▸) local implementation is run by default with these options: total buffer scattering subtracted, *q*-values output in Å^−1^, 7 Å as the envelope distance, keeping ligands but removing crystallization agents, replacing seleno­methio­nine with me­thio­nine, and using the experimental SAXS *I*(*q*) data provided to define the *q*-value grid. The ‘no random seed’ option is set for *WAXSiS* to support reproducibility. The *WAXSiS* calculation on the initial structure is always performed in ‘normal’ convergence mode, whereas for the NNLS preselected models, the default remains ‘normal’; however, an option of ‘quick’ convergence mode is provided (more details about the *WAXSiS* convergence modes can be found on the website https://waxsis.uni-saarland.de/help). For the *CRYSOL* and *WAXSiSI*(*q*) calculations, the solvent electron density is user selectable (in the ‘Load structure’ webpage), with the default set to 0.335 e Å^−3^, while *PEPSI-SAXS* does not use this entry. Calculated *I*(*q*) profiles are always scaled to the experimental data, using their associated standard deviation (SD) values to calculate the normalized χ^2^ of the scaling. Note all *I*(*q*) calculations are linearly interpolated to the *q*-values of the user-provided experimental data. When called, the *I*(*q*) calculators are requested to produce the same number of *q*-points as in the experimental data; however, most calculators have limits on the number of *q*-points, so interpolation is required to maintain identical *q*-values. The procedure for the NNLS selection of contributing structures is described in detail by Brookes *et al.* (2023 ▸). We note here that NNLS utilizes 1/SD weighting when SDs are available for *I*(*q*), and calculations are performed without and optionally with this weighting for *P*(*r*).

## 3. Website description

The website is organized as a number of sequential tasks, roughly divided into SAXS data and structure uploading, definition of flexible regions and generation of sequential conformations, preselection of a restricted number of model structures whose fast-calculated SAXS parameters add up matching the experimental data, and a final *WAXSiS*-based model selection against only the *I*(*q*) experimental data.

### 3.1. Website layout and login/registration page

The *SAXS-A-FOLD* website is conceived as a series of sections (‘Tabs’) on separate webpages, each one performing predefined tasks, all accessed from links in a common top bar, as shown in Fig. 1 ▸. The currently selected Tab will have its label changed to a bold font as shown in Fig. 1 ▸. Registration is required, and a Login/Registration module will pop up on accessing the webpage. A username and password must be entered, together with a valid e-mail address, which can be used for recovering a forgotten password and will be used by the server to send job completion messages. Registration is subject to webmaster approval, and the e-mail will not be shared outside the *SAXS-A-FOLD* system unless the user chooses to employ *CRYSOL*. After login, the main webpage will become available. On hovering the mouse on the section labels, explanation notes will pop up. To proceed, the Tab ‘Define project’ must be accessed first by clicking on its label.

### 3.2. Section 1: Define project

A project must be defined, allowing the user to keep track of past actions and to retrieve data already processed. Fig. 2 ▸ reports an example of project definition. A previously defined project can be also retrieved from this Tab. Pressing ‘Submit’ will either submit a new project or retrieve a previous one, enabling the user to proceed to the other sections. If an existing project name is entered, a pop-up message will appear asking the user to choose between ‘Erase previous results’ or ‘Keep previous results’. In the second case, the user can jump to any other sections containing previously generated results.

### 3.3. Section 2: Load SAXS

**Figure 3:**
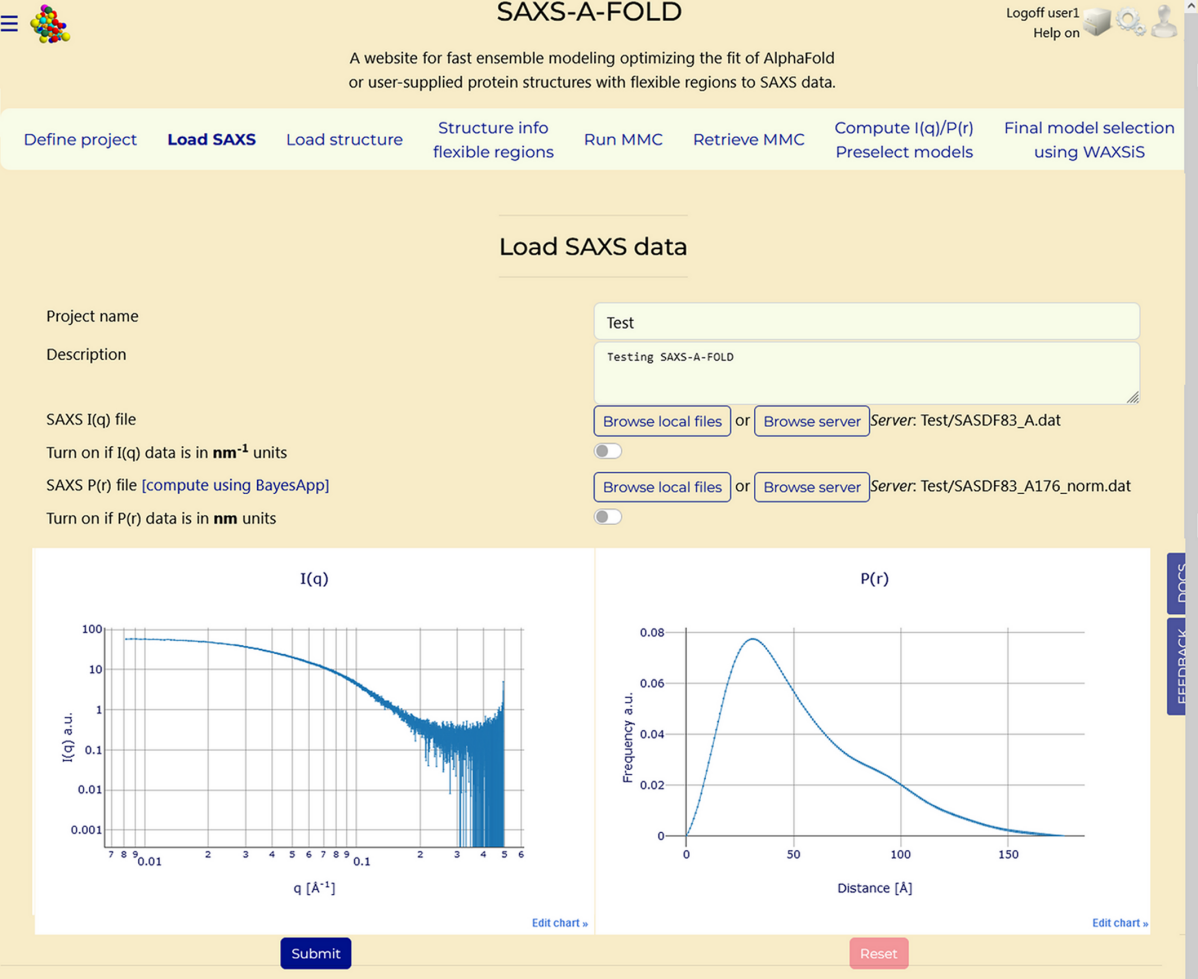
The Load SAXS Tab, with the *I*(*q*) and *P*(*r*) data loaded from the server.

Experimental [*i.e.I*(*q*)] and experimentally derived [*i.e.P*(*r*)] SAXS data must be uploaded and will be displayed and checked in this Tab, as shown in Fig. 3 ▸. Checks on uploaded data quality are planned but not yet implemented. The SAXS *I*(*q*) data are first uploaded, and if a corresponding *P*(*r*) is not already available, it can be generated by utilizing the link provided to the *BayesApp* website (Larsen & Pedersen, 2021 ▸; https://somo.chem.utk.edu/bayesapp). Both types of data can be loaded either from the user’s local directory (‘Browse local files’) or, if they were previously uploaded to the *SAXS-A-FOLD* website, from the stored data directory accessible via the ‘Browse server’ button. In the example shown in Fig. 3 ▸, the SASBDB SASDF83 *I*(*q*), corresponding to data acquired by Duarte *et al.* (2020 ▸) on *Homo sapiens* Bruton’s tyrosine kinase (mature protein residues 2–659), and its GNOM-derived *P*(*r*) (Brookes *et al.*, 2023 ▸) are shown using the *Plotly* interactive plotting utility. Users can specify if their scattering vector magnitude *q* units are in Å^−1^ or nm^−1^ [as derived from the relation *q* = (4π sin θ)/λ, see above] and if those of the distances *r* in the *P*(*r*) function are in Å or nm. Hovering the mouse on the load *I*(*q*) and *P*(*r*) buttons will show pop-up explanations of these options. However, all subsequent computations within *SAXS-A-FOLD* use Å^−1^ as the units for *q* and Å for *r*. Therefore, if the data are uploaded as nm^−1^ (and nm) they will be immediately internally converted to Å^−1^ (and Å).

### 3.4. Section 3: Load structure

**Figure 4:**
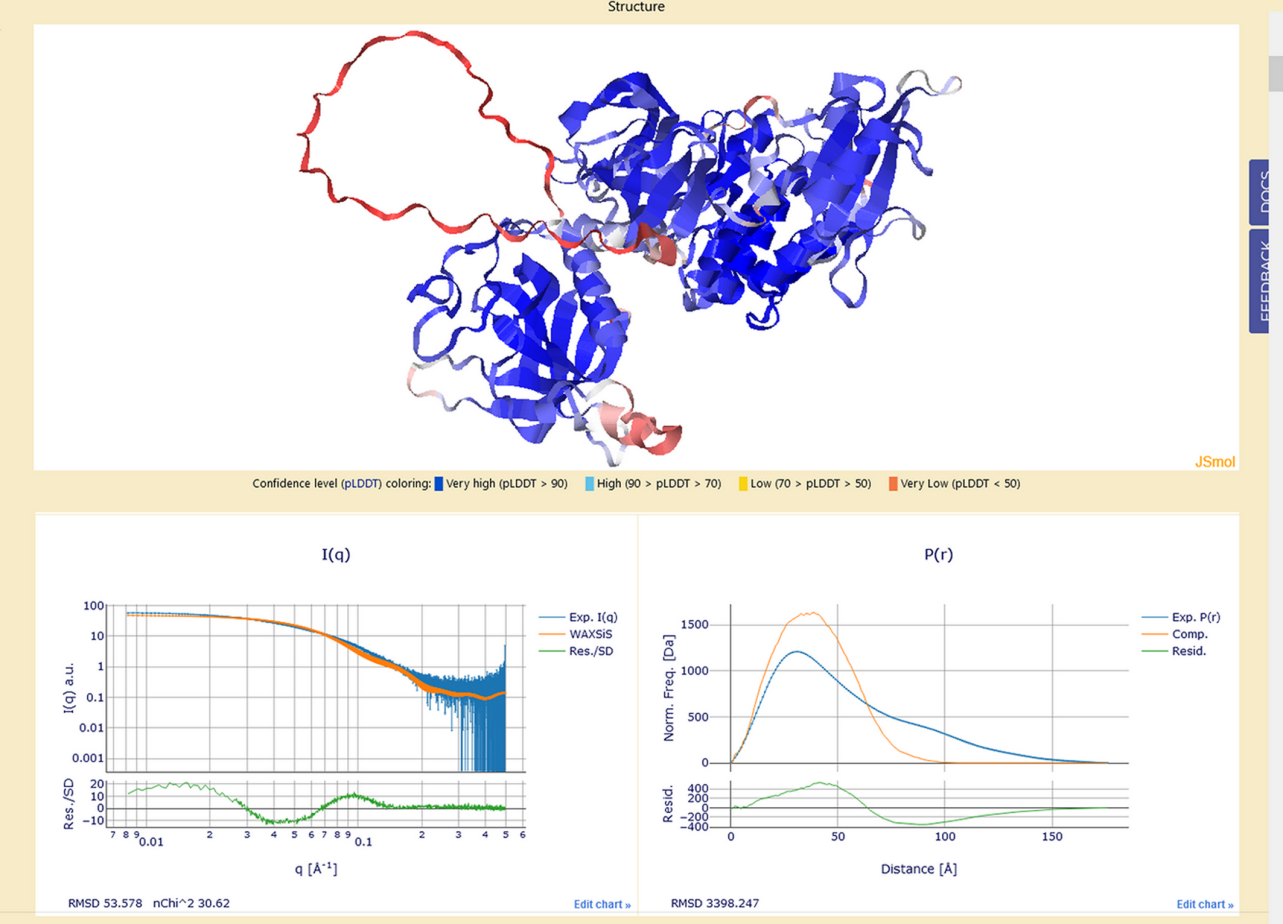
(Top) Graphical output of the ‘Load structure’ Tab, with the uploaded structure visualized by *JSmol* in ribbons mode and color-coded from red (low) to blue (high) according to the *AF*-provided confidence level values (below it, a link to the *AF* website describing the pLDDT is provided, followed by the color-coded pLDDT ranges). (Bottom, left) Experimental *I*(*q*) (blue) overlaid with that calculated by *WAXSiS* on the uploaded structure (orange), with the residuals/SDs of the scaling shown below it. (Bottom, right) Experimentally derived *P*(*r*) (blue) overlaid with that computed on the uploaded structure (orange), with the residuals shown below it.

The structure of this Tab and its basic operations and numerical results can be seen in Fig. S1 of the supporting information. If a structure had been already loaded in a previous session of the current project, it can be retrieved from the server. Otherwise, first a source for the structure must be selected from the ‘Select input source’ field. Here the mutually exclusive options are to upload a user-provided file (in PDB-, CIF- or mmCIF-format; see PDB-101: https://pdb101.rcsb.org/learn/guide-to-understanding-pdb-data/beginner%E2%80%99s-guide-to-pdbx-mmcif) or to directly load from the AF2 protein structure database (https://github.com/google-deepmind/alphafold/tree/main/afdb) or to directly load from a repository of partially curated AF2 structures (see Brookes & Rocco, 2022 ▸; https://somo.genapp.rocks). In the last two cases, the UniProt accession code must be entered in the field provided; typing part of it and pressing ‘Process’ will list the currently available (up to 25) entries sharing those characters, among which the required structure can be chosen. In all cases, the entry will be then automatically processed by a local installation of the *US-SOMO* program (see Brookes & Rocco, 2023 ▸; https://somoweb.genapp.rocks), but without the hydro­dynamic and circular dichroism computations. A progress bar reports the advancement of the computations, which in addition include the *I*(*q*) calculations using a *WAXSiS* local installation run in ‘normal’ convergence mode. In this example, for the *AF2* Q06187 structure comprising 10694 protein atoms and 44027 water atoms, the calculations took about 37 min. Messages relating to the various steps are printed below the progress bar. Once the calculations are completed, directly below the ‘Title’ and ‘Source’ fields (always populated if the entry comes from the *AF2* or our *AF2*-curated databases, otherwise depending on the content present in the uploaded PDB structure), a series of information and parameters are reported, including the mean *AF* prediction confidence level (predicted local distance difference test, ‘pLDDT’; Gomez & Kovalevskiy, 2024 ▸), molecular mass (Da), partial specific volume (cm^3^ g^−1^), theoretical hydration at pH 7 (g H_2_O per g protein), radius of gyration (*R*_g_, Å), and % α-helix and β-sheet content, as shown in Fig. S1. Below the parameter listing, the structure is displayed in ribbons mode via the interactive *JSmol* applet for molecular visualization [with the residues color-coded from red to blue in order of increasing *AF*-provided confidence level], followed by the *I*(*q*) and *P*(*r*) calculations superimposed on and scaled to the experimental curves, as shown in Fig. 4 ▸ [*P*(*r*) are displayed with the frequencies normalized by the structure’s calculated molecular mass, *i.e.* the total area under the interactive plot now corresponds to the structure’s molecular mass]. For *I*(*q*), the error-weighted residuals between the experimental and calculated curves are shown. For *P*(*r*), no error weighting is applied. Below the interactive plots, the RMSD of the scaling, and for *I*(*q*) also its normalized χ^2^ (‘nChi^2’), are reported. For the example shown in Fig. 4 ▸, note how the large oscillations in both residual plots indicate a poor fitting of the calculated versus the experimental and experimentally derived data, confirmed by the high RMSD and *n*χ^2^ values. At the bottom of the page, a complete listing of the *US-SOMO* processing output is shown, including identified di­sulfide bridges, the amino acid sequence in three- and one-letter codes, the overall amino acid composition, various physico-chemical parameters, and the log of the operations involved, including those relating to the *WAXSiS* processing (image not shown). From the *I*(*q*) and *P*(*r*) comparisons between experimental and calculated data, the user can judge if the agreement is satisfactory or if further action is needed to achieve a better fit. For the example shown in Fig. 4 ▸, especially in the *P*(*r*) plot, relevant differences between the experimental and the structure-derived data are evident and indicate a more extended shape for the protein in solution with respect to the *AF2* prediction (see Brookes *et al.*, 2023 ▸).

### 3.5. Section 4: Structure info/flexible regions

**Figure 5:**
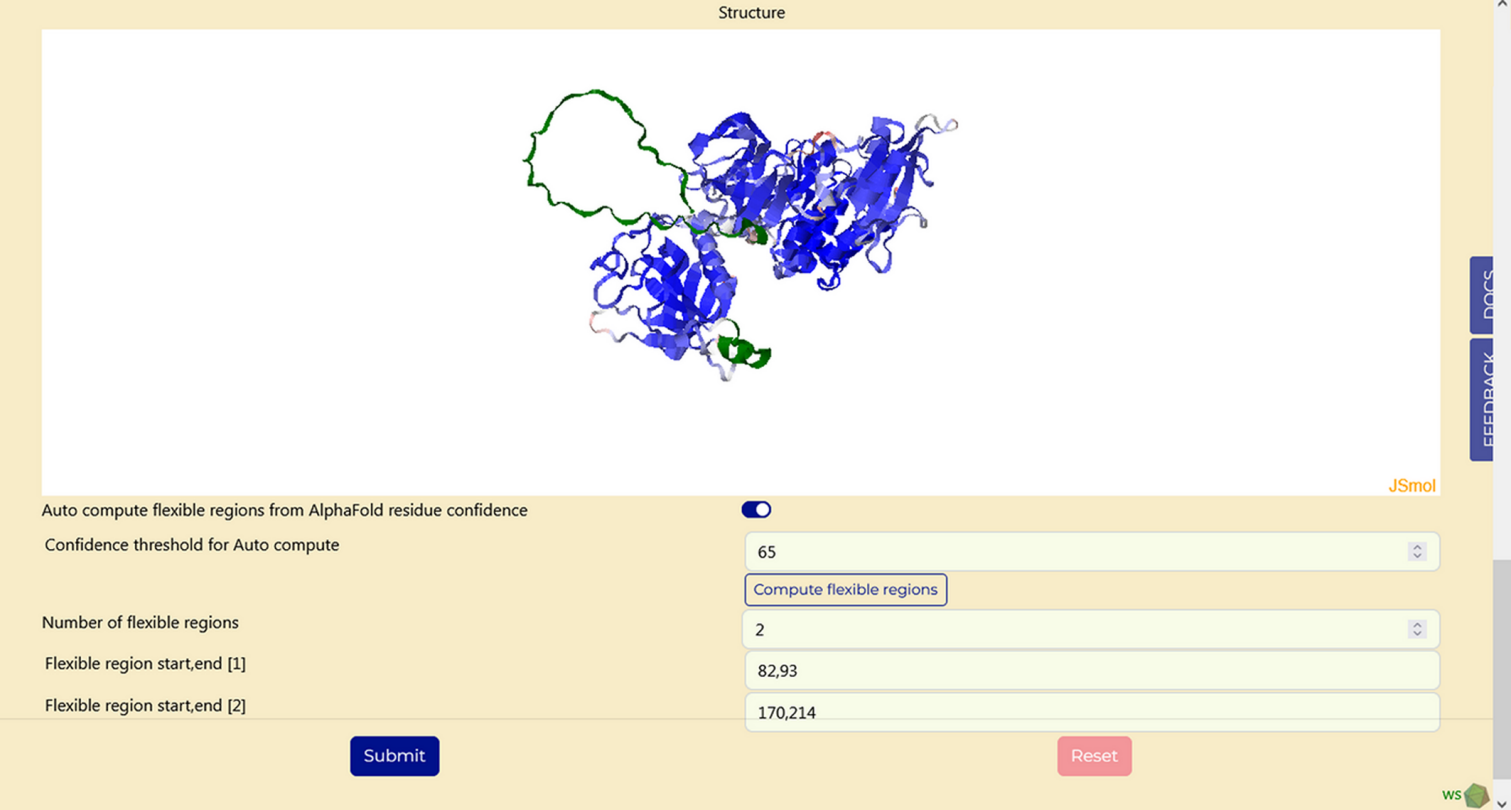
The lower half of the ‘Structure info/flexible regions’ Tab, showing the Q06187 *AF2* structure after checking the potentially flexible regions with a confidence level cut-off of 65. The two selected regions are color-coded in green in the *JSmol* graphical window.

This step is used to identify and enter flexible regions for the subsequent *MMC* step. Once the ‘Load structure’ step has completed, and if there are clear discrepancies between experimental and calculated *I*(*q*) and *P*(*r*) profiles, conformational expansion utilizing potentially flexible regions between structured domains/modules can be chosen by first hitting the ‘Structure info/flexible regions’ Tab. In this module, the previously computed protein parameters are first re-displayed, followed by the *I*(*q*) and *P*(*r*) plots (not shown). Below these plots, the structure is again shown, and below it an ‘Auto compute flexible regions from AlphaFold residue confidence’ option is present, which will operate only on *AF*-generated structures. A ‘Confidence threshold’ is introduced to explore sequences of at least five consecutive residues whose confidence level is below the chosen threshold (default: 60). Pressing ‘Compute flexible regions’ will generate a list of the amino acids stretches that have passed the threshold. These will be colored green in the *JSmol* updated graphical window, as shown in Fig. 5 ▸, obtained by increasing the threshold to 65.

Two sections were selected in this case. Users can either be satisfied with this output or explore different threshold values. If one region is deemed to be sufficient, and a threshold only selecting it cannot be found, the user can uncheck the auto-selection mode and manually enter a single flexible region with the starting and ending amino acid positions. In any case, this is the procedure that must be utilized if the uploaded structure does not come with associated confidence level values, first choosing the number of flexible regions and populating with the residue interval(s) the associated fields that will be displayed. For this example, a single region [170–210, following Brookes *et al.* (2023 ▸)] was chosen. Pressing ‘Submit’ will then record the chosen segment(s) for the Monte Carlo procedure that constitutes the next step in the *SAXS-A-FOLD* processing sequence.

### 3.6. Section 5: Run MMC

**Figure 6:**
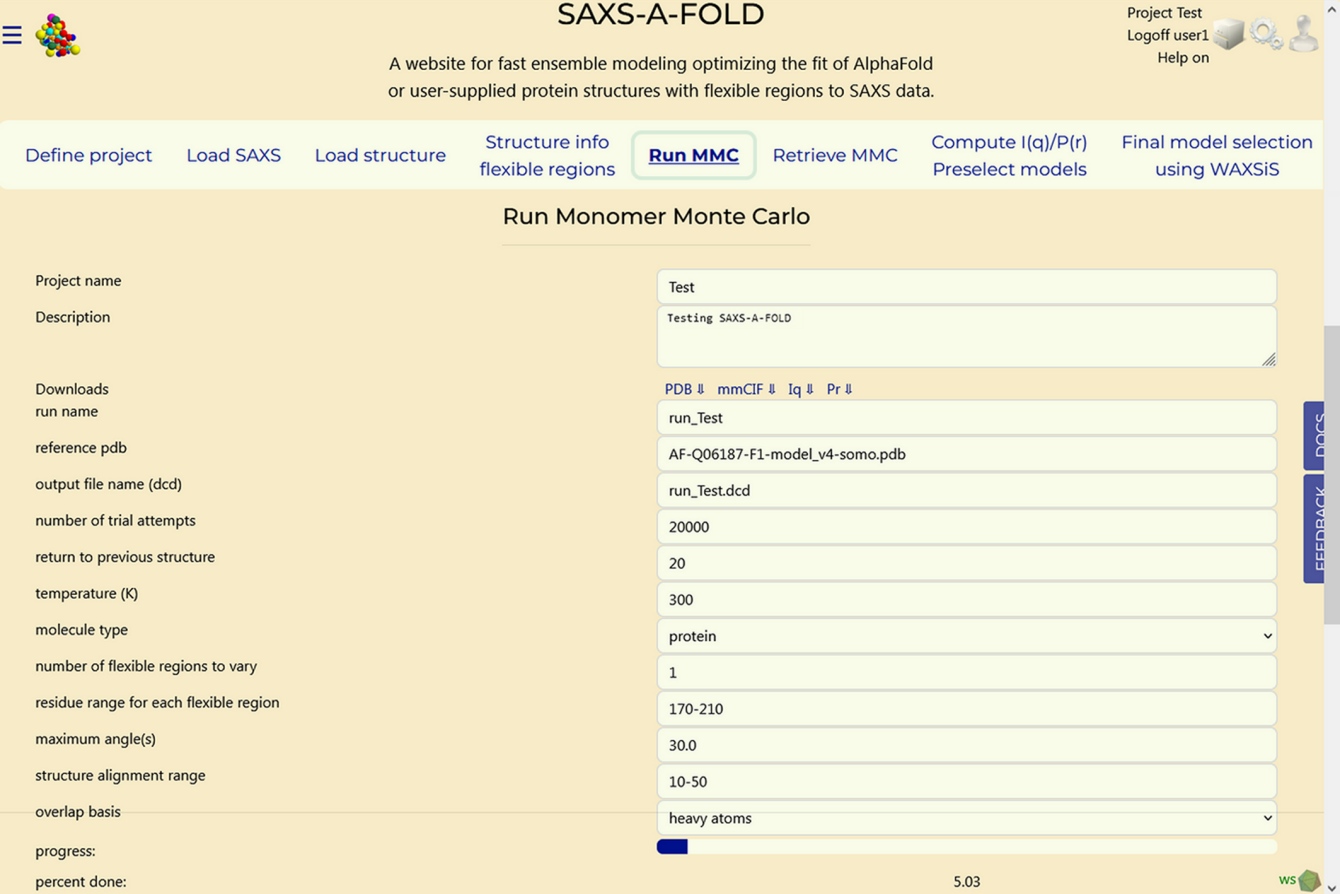
‘Run MMC’ Tab parameter setting section. The progress bar and percentage advancement will start to be updated once the run is launched by pressing ‘Submit’ (see Fig. S2).

This step produces an initial pool of models. A local implementation of the *MMC* procedure developed at NIST (Curtis *et al.*, 2012 ▸) is set and run in this Tab. First, the *MMC* run parameters must be set, as shown in Fig. 6 ▸. The various entries are automatically populated either with the settings determined in the previous tabs, like the ‘number of flexible regions to vary’ and their limits, or with currently fixed parameters, such as the ‘return to previous structure’ (set to ‘20’; after this number of failed step attempts, the program will reset to the current coordinates), the run ‘temperature’ (set to 300 K), the ‘molecule type’ (set to ‘protein’) and the ‘maximum angle(s)’ (set to 30°, which is the maximum angle that each torsion in each of the flexible regions can sample in a single move). The user-modifiable parameters are the ‘number of trial attempts’ (default: 50000; set to 20000 in this example), the ‘structure alignment range’ (the residue range used to spatially align all the *MMC*-generated models; it must be set to a non-flexible region, otherwise an error message will pop up) and the ‘overlap basis’ for the overlap checks used to reject structures with clashes (default: heavy atoms; other options include backbone heavy atoms, or all atoms if the structure also has hydrogen atoms defined).

The trajectory is saved as a binary DCD-formatted file, containing all the generated models, using the project’s name as its filename. Once all the fields have been properly set, pressing ‘Submit’ will launch the *MMC* run, whose progress is monitored by the bar and by the ‘percent done’ updating number (see Fig. 6 ▸, bottom).

As shown in Fig. S2, at the end of the *MMC* run, which in this case took about 30 min to complete, a graph of the *R*_g_ of all generated models and of the accepted models versus model number will be displayed, followed by a text summary where, among other data, the number of accepted models out of the total generated is reported.

### 3.7. Section 6: Retrieve MMC

**Figure 7:**
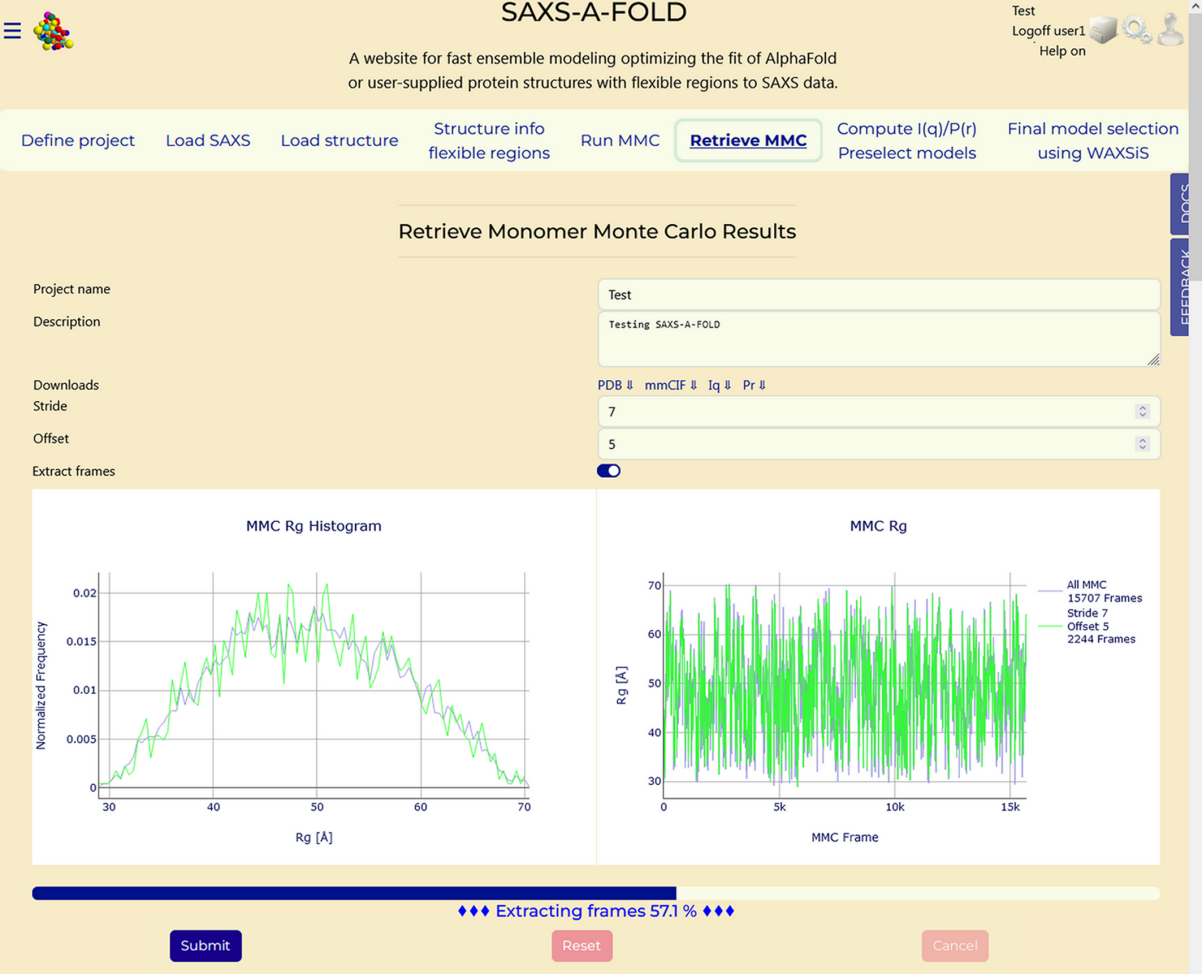
‘Retrieve MMC’ Tab. The progress bar and percentage advancement will start to update once the run is launched by pressing ‘Submit’. The graphs produced in a previous run – histograms of the *R*_g_ value frequency of the entire pool and that of the sub-selected pool (left), and the *R*_g_ values versus the frame (model) number for both pools (right) – are shown while a new run is being performed.

This step extracts models from the *MMC* pool to produce a reduced set. In this Tab a reduction of the *MMC*-generated models pool is performed in order to allow reasonable processing times during the *I*(*q*) and *P*(*r*) calculations. To ensure that the reduced model set is a faithful representation, with respect to the *R*_g_ distribution, of the entire *MMC* pool, the ‘Stride’ (default 10 frames) of the reduction can be changed prior to enabling the ‘Extract frames’ switch, as shown in Fig. 7 ▸. Pressing ‘Submit’ will then generate two plots, reporting on the left side histograms of the frequency of *R*_g_ values for the entire pool and that of the reduced pool, and on the right side the *R*_g_ values versus the frame (model) number for both pools (Fig. 7 ▸). It is also possible to change the starting model from which the stride is applied, by using the ‘Offset’ field (default: 0). Once a satisfactory histogram is attained by selecting the stride and the offset values, the ‘Extract frames’ switch can be enabled. On pressing ‘Submit’, the relatively long process of extracting the reduced pool of models from the large *.dcd file is started, monitored by the progress bar and its associated message, as shown in Fig. 7 ▸. For this example, a stride of 7 and an offset of 5 were chosen, and the extraction took 32 min.

### 3.8. Section 7: Compute P(r) and I(q), Peselect models

The penultimate step in *SAXS-A-FOLD* filters the reduced *MMC* pool to produce the set of preselected models. This stage generates the *I*(*q*) and *P*(*r*) curves for each model in the reduced pool, produced in the previous Tab, and applies an NNLS procedure to find the models whose calculated curves best add up in different proportions to match the experimental data. On selecting the ‘Compute I(q)/P(r), Preselect models’ Tab, if in a previous session this task had been already performed, its results will be shown (see Figs. S3 and S4), but they can be replaced by new sets either by changing the two options provided or by applying different reduction parameters in ‘Retrieve MMC’.

The two options relate to the choice of the fast *I*(*q*) computation method and if the *P*(*r*) NNLS fit should also be performed using the SDs associated with the experimentally derived curve. For the fast *I*(*q*) computations, currently *SAXS-A-FOLD* offers *PEPSI-SAXS* (Grudinin *et al.*, 2017 ▸) and, for academic users only, *CRYSOL* (version 3.2.1; Manalastas-Cantos *et al.*, 2021 ▸). The choice is made from the provided listbox, and if the *CRYSOL* option is chosen, a pop-up message will appear requesting confirmation of academic user status when pressing ‘Submit.’ Users can choose a single method or run both sequentially. The produced files will be marked accordingly. A second switch, ‘Advanced options’, will provide additional controls in a future release.

**Figure 8:**
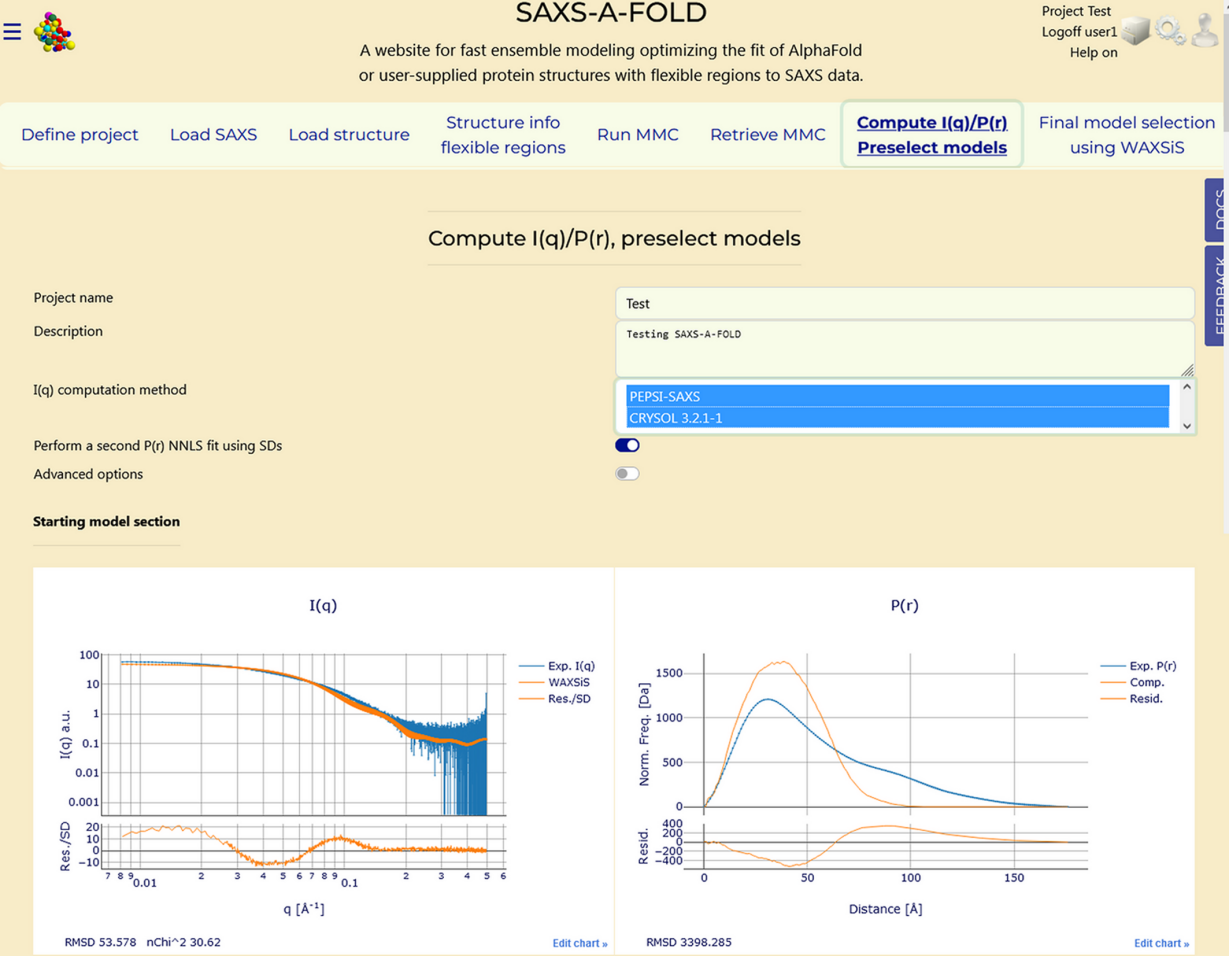
‘Compute *I*(*q*)/*P*(*r*), Preselect models’ Tab. Shown is the initial section where the choice for the *I*(*q*) computation method is done, with both currently available options having been selected. Also shown is the activated switch for performing a second *P*(*r*) NNLS fit using the original associated SDs. Below the settings, in the ‘starting model section’ the *I*(*q*) and *P*(*r*) graphs with the experimental curves and those calculated for the initial structure shown previously in Fig. 4 ▸ are reproduced. See Figs. S3 and S4 for the results.

In the case of a first-time run, only the interactive plots present in the ‘Starting model section’ of Fig. 8 ▸ will be shown These contain the experimental *I*(*q*) and experimentally derived *P*(*r*) plots with superimposed curves calculated on the starting structure and their residuals, as shown in Fig. 4 ▸. The graphs in the other sections will start to appear once the ‘Submit’ button is pressed, beginning with those in the ‘P(r) results section’ (see Fig. S3). To overcome browser limitations, static images instead of interactive plots are produced for the calculations on all models in the reduced pool and updated in blocks of curves (currently 50). A progress bar and associated messages are provided below the updated images. Once the *P*(*r*) calculations are completed (including the NNLS fit, see below), the *I*(*q*) computations will start, with the results displayed in the ‘I(q) PEPSI-SAXS results section’ or in the ‘I(q) CRYSOL results section’. If both methods were selected, the *PEPSI-SAXS* results will be processed and shown first (see Fig. S4). In this example, which used both methods for the *I*(*q*) computations, the entire process generating 2 × 2244 *I*(*q*) and 2444 *P*(*r*) curves took approximately 5 h.

After each *P*(*r*) or *I*(*q*) curve computation process is completed, the NNLS procedure is applied. The results are displayed sequentially, with each model’s number and percentage contribution to the fit listed below the respective interactive plot, as shown in Figs. S3 and S4. In this example, the *P*(*r*) NNLS fit using SD weights was also computed. Note that a few models were selected in common by each method. Below each set of NNLS model selection results, links are provided for downloading the complete sets of generated curves as .csv files.

### 3.9. Section 8: Final model selection using WAXSiS

The final step in the *SAXS-A-FOLD* procedure is to generate a set of *I*(*q*) curves for each preselected model using our local implementation of the more physically advanced but computationally intensive *WAXSiS* program (Chen & Hub, 2014 ▸; Knight & Hub, 2015 ▸) and to perform a final NNLS-based selection of best-contributing models matching the experimental *I*(*q*) data.

**Figure 9:**
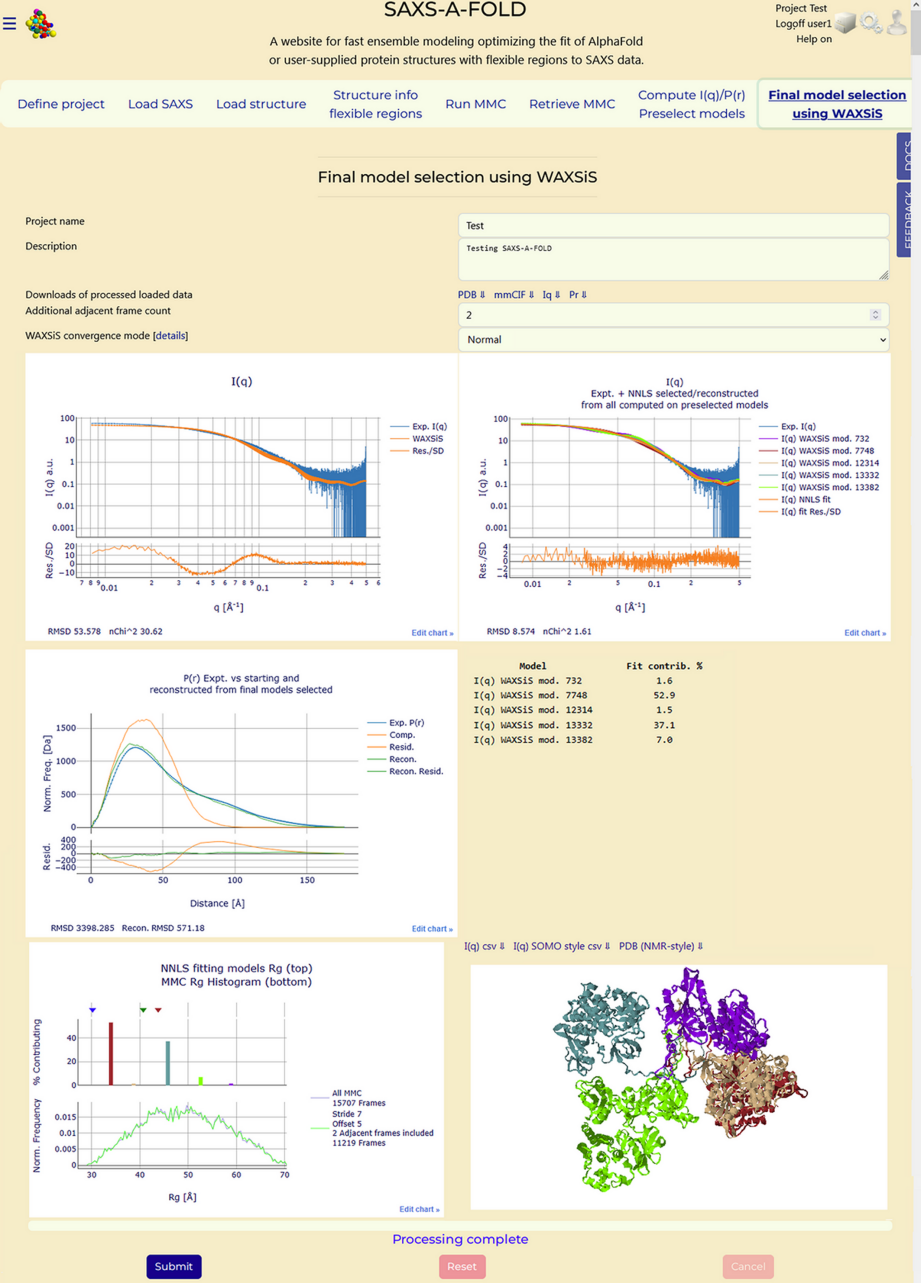
‘Final model selection using WAXSiS’ Tab. In the ‘Downloads of processed loaded data’ field, there are links for downloading the starting structure and the *I*(*q*) and *P*(*r*) calculated from it, together with the corresponding experimental data. The ‘Additional adjacent frame count’ field comes next (2 in this case), followed by the ‘WAXSiS convergence mode’ (‘Normal’ here). The experimental *I*(*q*) is shown (blue) in the top-left graph, overlaid with that calculated by *WAXSiS* on the starting structure (orange, with the residuals/SDs displayed below it), while in the top-right graph it is overlaid with the NNLS results (contributing models, various colors; orange is the fitted curve, with the residuals/SDs also displayed). Below these graphs, on the right there is a list of the NNLS-selected models with their percentage contribution, and on the left a *P*(*r*) plot with the experimentally derived curve (blue), that calculated on the starting model (orange) and a reconstructed curve using the *P*(*r*) computed on the structures selected by the *WAXSiS* NNLS, weighted by the percentage contribution. The residuals for the starting model and reconstructed *P*(*r*) versus the experimentally derived curves are shown below the main graph. Below the *P*(*r*) graph, two histogram plots are shown. On top is that of the percentage contribution versus *R*_g_ values for the selected models (bars, hovering the mouse will reveal model number, percentage and *R*_g_), with three inverted triangles reporting the *R*_g_ values of the starting structure (blue), the weighted average of the selected models (green) and that calculated from the experimentally derived *P*(*r*) (brown). In the second plot, the *R*_g_ value frequency of the entire pool and of the sub-selected pool are reproduced. At the bottom of the page there is a *JSmol* window with the NNLS-selected models, color-coded as in the NNLS fit and histogram plots.

The models preselected in the previous step are known to this module. In addition, since a sub-selection stride on the initial pool of *MMC*-generated models was applied, and some potentially better-fitting models were not even considered, adjacent models flanking (on both sides) those preselected in the previous step can be added at this stage. This is a new feature that was not present in the Brookes *et al.* (2023 ▸) study and which can be considered a further refinement of that procedure. The number of adjacent models for each pre-selected model can be entered in the ‘Additional adjacent frame count’ field (see Fig. 9 ▸), but it cannot be greater than half the sub-selection stride value. In the example shown here, two adjacent models on either side of the pre-selected models were added. The ‘WAXSiS convergence mode’ field allows users to select ‘quick’ or ‘normal’ from a pull-down menu (see *Methods*[Sec sec2] for a description of these two alternative options). In ‘quick’ mode *WAXSiS* takes about half as much time as in ‘normal’ mode, and it might be advisable to perform a first run in this mode (also without adjacent models) before refining the results using the ‘normal’ mode. For this test, we performed two *WAXSiS* runs at the end, both with two adjacent models (for a total of 115 models), one in ‘quick’ and the other in ‘normal’ mode. The latter took approximately 3 days and 14 h to complete.

On pressing ‘Submit’, the user is provided with an estimate of the time that *WAXSiS* will need to complete all the calculations, based on the time that it took to compute the *I*(*q*) curve for the starting structure in the ‘Load structure’ module, and is asked for confirmation to proceed. Once the procedure is started, this time estimate is updated after each *WAXSiS* computation is completed, as different models can require shorter or longer calculations, depending especially on the size of the generated water box. More information on the *WAXSiS* operations and advancement is provided in the text area at the bottom of this Tab, including a list of all models that will be processed (not shown).

On completion of the *WAXSiS* calculations, a final NNLS fit is done on all curves, including the one for the starting model, against the experimental *I*(*q*) data. The results are reported in a new interactive plot positioned on the right side of the plot with the initial comparison between the experimental *I*(*q*) and that calculated for the starting model, as shown in Fig. 9 ▸. This new plot contains, scaled to the experimental *I*(*q*) curve, the *WAXSiS*-calculated *I*(*q*) profiles for each NNLS-selected model (with their associated SDs) and, as the last plotted curve, the reconstructed *I*(*q*) based on the percent contribution of each computed *I*(*q*) curve (with the propagated SDs). By clicking on each entry ‘line’ in the legend, they can be turned on and off in the interactive plot. Below this plot, the NNLS-selected models are listed, along with their percentage contribution. To the left side of the model list, a new *P*(*r*) plot is displayed, with the experimentally derived *P*(*r*) overlaid with that of the starting model and a composite one produced by summing those of the individual *WAXSiS*-selected models, weighted by their percentage contributions. This plot (Fig. 9 ▸) is meant to confirm that the models selected on the *I*(*q*) data can also reasonably match the *P*(*r*) data, albeit with a worse fit mainly due to the *P*(*r*) computations being on the dry structures. Nevertheless, a sixfold improvement in the RSMD can be seen for the reconstructed *P*(*r*) plot in Fig. 9 ▸. Both the entire dataset of *I*(*q*)s and the selected models (in an NMR-style single file) can be downloaded using the links provided. Below the *P*(*r*) plot, two superimposed histogram plots are shown (Fig. 9 ▸). The top plot reports the percentage contribution versus *R*_g_ values for the selected models (bars, same color as in the NNLS fit plot) and, marked by three inverted triangles, the *R*_g_ values of the starting structure (blue), the weighted average of the selected models (green) and that calculated from the experimentally derived *P*(*r*) (brown). Hovering the mouse over the inverted triangles will reveal details including *R*_g_ values and over the bars will reveal model number, percentage and *R*_g_. In the second plot, the *R*_g_ value frequency of the entire pool and of the sub-selected pool is again reproduced.

Finally, to the right side of the histogram plots, all the selected models are shown, spatially aligned on the previously chosen sequence, in an interactive *JSmol* viewer. Each model is by default represented in ribbons mode and colored as the corresponding curve in the NNLS fit and bar in the histogram plot. Users can access a full range of viewing options, including showing only a particular model, by right-clicking on the viewer.

In Table 1 ▸, we have collected all the *MMC* models selected by the various procedures (column 1), with their *R*_g_ values (column 2) and the percentage found by the NNLS fits (all other columns), including the adjacent models that were selected by the final *WAXSiS*-based NNLS fits. Some *WAXSiS*-based NNLS fits, including either of the *I*(*q*) fast method selections coupled with both the *P*(*r*) selections, are reported in Table 1 ▸ (columns 7 and 8). At the bottom, the *n*χ^2^ of the NNLS fits are reported [except for the *P*(*r*) fits]. The first thing to notice is that every preselection operation contributed models either in common with others or that were not considered by other methods. While a few common models were selected by the two *P*(*r*) selections (Table 1 ▸, columns 3 and 4), the two *I*(*q*) fast methods selected completely different models (Table 1 ▸, columns 5 and 6), underscoring that it is advisable to run both methods. When the *WAXSiS*-based selection was performed on the models selected by the two *P*(*r*) and a single *I*(*q*) fast method, several adjacent models were selected, but only one in common (model No. 9191 with ∼18 and ∼15% contributions; Table 1 ▸, columns 7 and 8). This already confirms that including the adjacent models could improve the selection procedure. Finally, the *WAXSiS*-based NNLS fits, including all preselected models and their adjacent models, one in ‘quick’ and the other in ‘normal’ mode, produced two apparently completely different sets of contributing models (Table 1 ▸, columns 9 and 10). While the *n*χ^2^ of the run done in ‘quick’ mode is lower than that of the run done in ‘normal’ mode, this could result by lower accuracy *I*(*q*) curves just stochastically producing a better fit. Computing the [〈
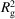
〉]^0.5^ of the two sets (multiplying each squared *R*_g_ value by its fractional contribution, and taking the square root of the sum) yielded 40.1 and 40.2 Å, respectively, which is lower than both the experimentally derived values from Guinier and *P*(*r*), 41.3 and 43.8 Å, respectively, as was also observed in the Brookes *et al.* (2023 ▸) study.

We can now compare the resulting models obtained in both *WAXSiS* modes for the Q06187/SASDF83 system in this test run of the *SAXS-A-FOLD* server with those presented by Brookes *et al.* (2023 ▸), notwithstanding several relevant differences. To begin with, the *MMC* procedure is stochastic; therefore different runs, even with the same overall starting parameters, will yield different sets of models. This is shown by the fact that in the Brookes *et al.* (2023 ▸) work the same number of trial attempts (20000) using the same flexible region (170–210) yielded 14582 structures, while 15707 were obtained here. It was, therefore, pointless to use the same stride, and we elected to generate a larger pool of reduced models, 2244 versus 972 used by Brookes *et al.* (2023 ▸), to enhance the search for best-fitting models. Secondly, two fast *I*(*q*) calculators were used here, *PEPSI-SAXS* and *CRYSOL* (version 3.2.1), while Brookes *et al.* (2023 ▸) only used *CRYSOL* (version 2.8) (with a test of version 3.2 with dummy waters added as a final comparison). Third, we also included two models before and after the ones selected by the two *P*(*r*) and the two fast *I*(*q*) NNLS-based selections. Fourth, the final *WAXSiS*-based selection was done in the ‘quick’ and ‘normal’ modes, whereas Brookes *et al.* (2023 ▸) used the ‘thorough’ option.

**Figure 10:**
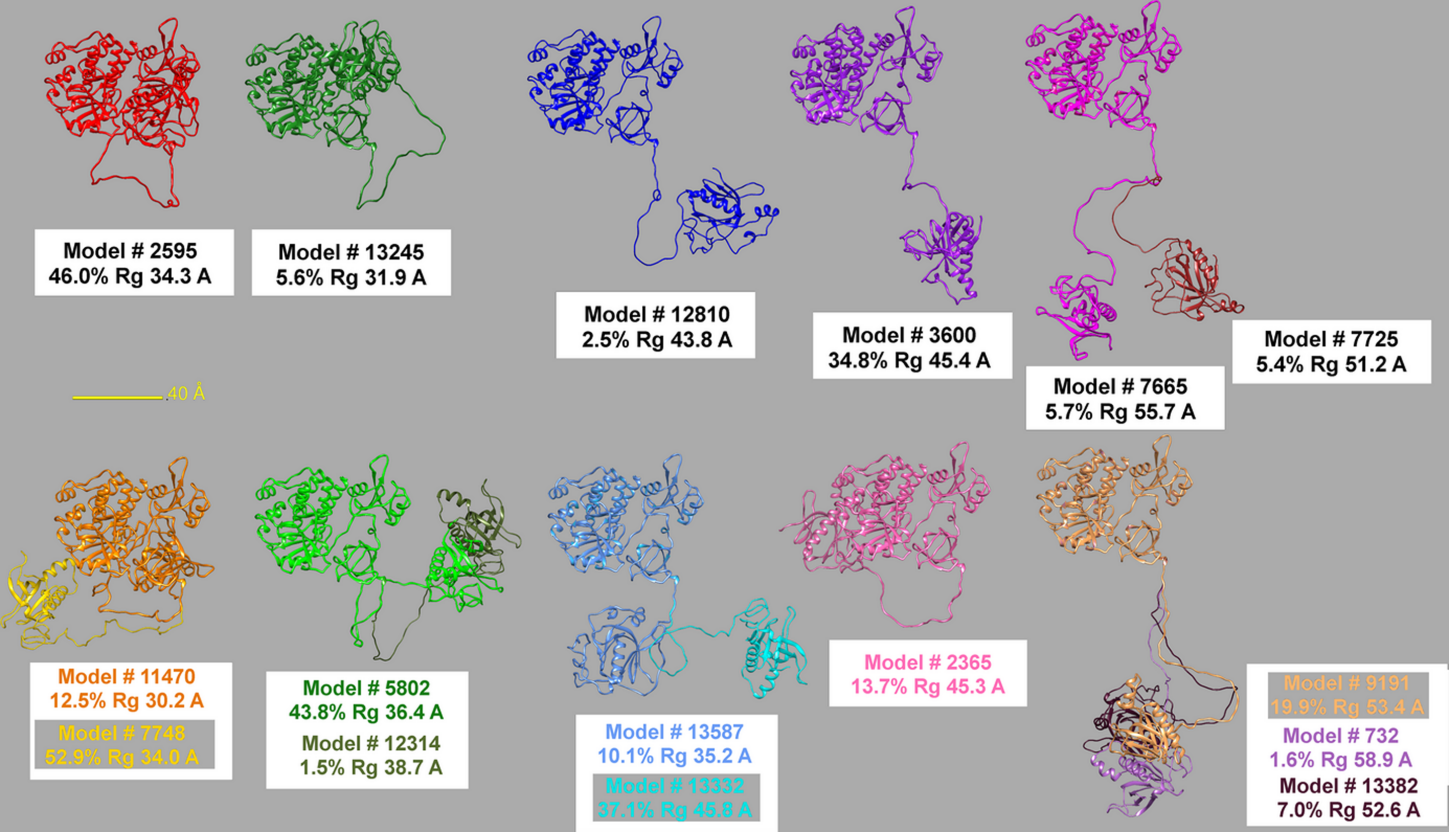
Comparison between the models selected for the Q06187/SASDF83 system in the Brookes *et al.* (2023 ▸) study (top row) *and* in the present study (bottom row). Models were all superposed using the C-terminal 218–659 sequence and rotated by the same amounts along the same axes to maximize their similarities. The bottom row reports the models NNLS-selected using *WAXSiS* run in ‘quick’ mode (models 11470, 5802, 13587, 2365 and 9191) and in ‘normal’ mode (models 7748, 12314, 13332, 732 and 13382).

In Fig. 10 ▸, we show a graphical comparison of the models that were obtained. In the top row, all the final models selected in the Brookes *et al.* (2023 ▸) study are displayed after superposition using the C-terminal 218–659 sequence, followed by a lateral translation. Two very similar models are left superposed (far-right models). In the bottom row, the two *SAXS-A-FOLD*-produced sets of models (‘quick’ and ‘normal’ *WAXSiS* runs) are then shown, first superposed on the Brookes *et al.* (2023 ▸) models on the same sequence according to a rough similarity check, and then translated downwards. All models are color-coded and labeled with their number, percentage contribution and *R*_g_ value. In the bottom row, the first label belongs to the ‘quick’ run and the other to the ‘normal’ run. Several models are quite similar, no one identical, as expected, and some are clearly different while having almost identical *R*_g_ values. Overall, these models confirm the findings of Brookes *et al.* (2023 ▸) that for this system the protein seems to assume a range of conformations in solution that on average are significantly more extended than the starting *AF*-predicted structure, although two dominant conformations, one close to the starting one, the other clearly more extended (see the histogram in Fig. 9 ▸), appear to be present.

## 4. Discussion and conclusions

We have described here a new public-domain website implementing the procedure developed by Brookes *et al.* (2023 ▸) to complement with SAXS data the structures predicted by *AF* (Jumper *et al.*, 2021 ▸; Abramson *et al.*, 2024 ▸) or composed by modules/domains solved experimentally, both having significant portions of unstructured and potentially flexible segments. Intrinsically disordered proteins can also be studied, provided that a basic set of coordinates is available. SAXS experiments report on the time and ensemble average of the structures present in solution, and thus are well suited to verify predictions of the conformational states adopted by (bio)-macromolecules (see Koch *et al.*, 2003 ▸). The *SAXS-A-FOLD* website (https://saxsafold.genapp.rocks) has been conceived to offer a streamlined sequence of actions culminating in the production of ensembles of models whose weighted calculated SAXS profiles can best match the experimental data. These ensembles can either represent defined structural conformers that can be stably populated and separated by low energy barriers or be indicative of true flexibility resulting in an extended range of transient conformations. More local conformational variability instances, such as extended side chain wobbling, are not dealt with specifically in *SAXS-A-FOLD*, as their contribution will likely be second order and would be more in the realm of full MD simulations. As a caveat, we also point out that there are no provisions within *SAXS-A-FOLD* to deal with incorrect or incomplete starting structures, either produced by *AF* or similar AI methods or experimentally solved, the burden of this task falling solely on the users.

The *SAXS-A-FOLD* operational framework performs a conformational expansion of the starting structure, varying, for the segments of potential flexibility, the torsion angles in the allowed Ramachandran regions along the protein backbone, using the *MMC* procedure developed at NIST (Curtis *et al.*, 2012 ▸; Perkins *et al.*, 2016 ▸). This can generate tens of thousands of sequential models, and it would be impractical to compute the SAXS profiles for each one, even using fast, more approximate methods. Therefore, the *SAXS-A-FOLD* strategy is to apply a reducing stride at a chosen interval –verifying, by comparing the *R*_g_ distribution, that it is representative of the entire *MMC*-generated pool – and calculate for the resulting reduced pool of models their *I*(*q*) versus *q* profiles in reciprocal space using either *PEPSI-SAXS* (Grudinin *et al.*, 2017 ▸) or, for academic users only, *CRYSOL* (version 3.2; Manalastas-Cantos *et al.*, 2021 ▸). Both are relatively fast methods. In addition, the real space pair-wise distance distribution function *P*(*r*) versus *r* is computed for the same set of models. Both datasets are then subjected to non-negatively constrained least squares selection against their experimental or experimentally derived counterparts, leading to the identification of a restricted number of models for which the sum of their weighted *I*(*q*) or *P*(*r*) profiles provides the best match. For *P*(*r*), a second set of models can be identified by including SDs as weights in the NNLS procedure. The SDs are computed during the derivation of the *P*(*r*) from the experimental *I*(*q*) data, although their reliability is still a subject of discussion. The *I*(*q*) of each model (some of which can be selected by all methods) in this filtered, reduced pool is then computed locally by the more physically advanced but computationally intensive *WAXSiS* method, which uses a short MD run with explicit solvent (Chen & Hub, 2014 ▸; Knight & Hub, 2015 ▸). The NNLS procedure is applied again on these sets of *WAXSiS*-calculated *I*(*q*) profiles against the experimental data. As shown by Brookes *et al.* (2023 ▸), notwithstanding the inherent approximation in the generation of the *P*(*r*) profile from the original *I*(*q*) data, and neglecting the hydration contribution in the calculation of the *P*(*r*) from atomistic models, performing an NNLS selection including them increases the number of contributing models in the final *WAXSiS*-based selection. Note that the NNLS algorithm guarantees results containing the best fitting, in the Euclidean norm, non-negatively constrained linear combination of models (Lawson & Hanson, 1995 ▸). From this, one can conclude that, if any models in the pool are ill-fitting, they will be filtered out at the final NNLS stage. It should be also pointed out that the search for the best models in *SAXS-A-FOLD* is not exhaustive, and the subset of models found to best fit the data can be taken as representative of the potential conformations sampled by the studied structure.

The above-described features distinguish *SAXS-A-FOLD* from other available methods where SAXS data are used in conjunction with a starting structure to produce models better fitting the experimental data. For instance, the widely used *EOM* (*Ensemble Optimization Method*) in the *ATSAS* suite (Tria *et al.*, 2015 ▸; Franke *et al.*, 2017 ▸; https://www.embl-hamburg.de/biosaxs/eom.html) generates a large number of random conformations from an initial model that is composed of structured portions joined by flexible linkers and then uses a genetic algorithm to select a number of conformers whose SAXS *I*(*q*) profiles, calculated by *CRYSOL*, best match the experimental data. *EOM* can use linkers not defined at the atomic level (dummy residues) and can apply other constraints presently not available in *SAXS-A-FOLD*. However, *SAXS-A-FOLD* uses both *I*(*q*) calculated by two fast methods (*CRYSOL* and *PEPSI-SAXS*) and *P*(*r*) calculated on the dry structures to pre-screen the ensemble of sequentially generated conformers. It then applies the *WAXSiS* advanced *I*(*q*) calculation method to finally select the ensemble of models best matching the experimental SAXS *I*(*q*) profile.

Another server using SAXS data to screen for conformational ensembles is *AllosMod*–*FoXS* (https://modbase.compbio.ucsf.edu/allosmod-foxs), which is a combination of *FoXS* (Schneidman-Duhovny *et al.*, 2010 ▸) and *AllosMod* (Weinkam *et al.*, 2012 ▸). *AllosMod* samples protein conformations using MD methods, while *FoXS* performs rigid body modeling of SAXS profiles. In the combined server the *AllosMod*-generated conformations are directly fed to *FoXS* for the computations of the *I*(*q*) profiles. In respect to the current implementation of *SAXS-A-FOLD*, *AllosMod*–*FoXS* can handle a wider range of structures, including RNA, DNA and carbohydrates, but relies only on the *FoXS* method to calculate the *I*(*q*) profiles and the ensemble evaluation is not as streamlined.

The *SAXS-A-FOLD* website is conceived as a series of web pages, all accessible from links (‘Tabs’) always present on each page. In this first *SAXS-A-FOLD* release, users can start by defining a project and then upload their SAXS *I*(*q*) experimental data and its derived *P*(*r*) in the next Tab. In the absence of the latter, a link is provided to the *BayesApp* website (Larsen & Pedersen, 2021 ▸; https://somo.chem.utk.edu/bayesapp) for its calculation. The starting structural model is uploaded in another Tab, either directly from the *AF2* database (https://alphafold.ebi.ac.uk) or from a repository of partially curated *AF2* structures (https://somo.genapp.rocks), or user-provided by direct upload (structures previously uploaded can be also retrieved from the *SAXS-A-FOLD* server). *SAXS-A-FOLD* immediately performs a series of calculations on the uploaded model structure, providing a series of physico-chemical and structural parameters and computing its *P*(*r*) and *I*(*q*), the latter using *WAXSiS*. The choice of the potentially flexible region(s) is done in the subsequent Tab, and for *AF* models it can be informed by the confidence level provided for each residue. This choice is automatically forwarded to the ‘Run MMC’ Tab, where all the parameters governing the run are shown and some can be modified, others being currently fixed to ‘standard’ default values. Once this somewhat long step (depending on the number and size of the flexible regions and on the number of trial attempts) is completed, its results can be shown in the ‘Retrieve MMC’ Tab, where the sub-selection stride and offset can be tested to ascertain that the resulting histogram of *R*_g_ values is representative of the general distribution of *R*_g_ values in the complete *MMC*-generated pool of models. Once a satisfactory agreement is reached, the reduction can be performed, extracting from the large *MMC*-generated model file only the reduced ones. At this stage, *SAXS-A-FOLD* is ready to compute the *P*(*r*) and *I*(*q*) of all reduced models in a dedicated Tab, where progress is monitored in real time. On completion, the NNLS fitting procedure will automatically take place, with the results presented graphically and numerically in terms of the goodness of the NNLS fits, contributing models (numbered according to their position in the original *MMC* output), their percent contribution to the NNLS fit, and their individual *P*(*r*) and *I*(*q*) profiles. Finally, in the last Tab *WAXSiS* will calculate the *I*(*q*) profile of all the NNLS-selected models and, if entered, the number of adjacent models in the original *MMC* run output. This new feature, with respect to the Brookes *et al.* (2023 ▸) work, was conceived to potentially ‘capture’ models missed by the preselection stride but that, in principle, could better contribute to the NNLS fitting procedure. However, using this option will noticeably increase the computing time required, which is projected according to the initial *WAXSiS* run time, and it is updated as more runs are completed. Once all *I*(*q*) calculations are done, a final NNLS fit is performed, with its results again displayed both graphically and numerically. The selected models are also visualized at this stage. All the results can be downloaded in appropriate formats.

*SAXS-A-FOLD* was tested using one of the *AF2* structures and SASBDB data used by Brookes *et al.* (2023 ▸), Q06187 and SASDF83. The results produced a similar outcome, both in terms of the shapes of models selected as producing the best fits in reciprocal space to the experimental *I*(*q*) data and in terms of their *R*_g_ values. The use of all preselection methods and of the adjacent models appears to have provided the best results. The selected models are indeed representative of a range of conformations that this protein evidently explores in solution. They could be taken as a basis for more advanced, all-atom, explicit solvent MD simulations. One last note: we have also explored adding a second potentially flexible region on the basis of the *AF* confidence levels, residues 82–93 (see Fig. 5 ▸) and increasing the number of trial attempts to 30000. Not surprisingly, many more models were selected, with the final *WAXSiS*-based NNLS fit having an *n*χ^2^ of 1.163, better than all other cases (see Fig. S5). Further analysis of this run is, however, outside the scope of this work.

A number of further developments are already being planned. At the level of the user-provided SAXS data, a series of checks on their quality will be implemented, and introducing additional features potentially limiting the *q*-range used and/or performing re-binning operations is being considered. Text and figures containing reports will be automatically generated and will be downloadable, which will include data from each of the separate tasks present in *SAXS-A-FOLD*. As the current version can deal only with single-chain protein structures with no prosthetic groups, an effort will be directed to overcome this limitation, mainly at the generation of the pool of models using more advanced Monte Carlo methods. This could allow expansion of the field of accepted types of structures to multi-chain proteins and complexes with other kinds of bio-macromolecules, such as single- and double-stranded nucleic acids. The *WAXSiS* ‘thorough’ option could also be added, depending on the computer power available. Note that although the current screenshots throughout this paper represent its state as of this writing, the ‘DOCS’ tab and tutorial will be regularly updated to reflect the current state.

In conclusion, we believe that the *SAXS-A-FOLD* website could become a useful tool, joining the expanded universe of predicted/solved, well defined structural units linked by unstructured regions with the foreseeable increase of meso-resolution SAXS data, exploring their behavior under more physiologically relevant solution conditions. The resulting pool of structural models could then be further enhanced by performing more advanced but computationally very intensive all-atom MD simulations with explicit solvent, with the ultimate goal of providing a deeper insight into their structure–function relationships.

## Supplementary Material

Supporting figures. DOI: 10.1107/S1600576725003590/ju5083sup1.pdf

## Figures and Tables

**Table 1:** Models selected by NNLS from the data produced by the various methods, and their percentages, for the AF-Q06187/SASDF83 system used as a test of *SAXS-A-FOLD*

Model No.	* R*_g_ (Å)	*P*(*r*) (%) (no SD weighting)	*P*(*r*) (%) (SD weighting)	*PEPSI-SAXS* (%)	*CRYSOL* 3.2.1%	*WAXSiS* (%) [*P*(*r*) + *PEPSI*]	*WAXSiS* (%) [*P*(*r*) + *CRYSOL*]	*WAXSiS* (%) all ‘quick’	*WAXSiS* (%) all ‘normal’
732	58.9								1.6
734	58.1	6.9		1.9					
1385	41.9				13.0				
2365	45.3			2.8		19.5		13.7	
2379	45.1	10.7	6.5		2.2		1.7		
5445	31.8	5.7			26.3		11.9		
5446†	29.6						2.9		
5802†	36.4						51.5	43.8	
7748	34.0		48.7		29.1				52.9
9036	41.8		23.7						
9043	43.9				3.7				
9057	46.6		5.1				17.0		
9190	54.0	7.4	2.4						
9191†	53.4					17.8	15.0	19.9	
11470†	30.2							12.5	
11471†	30.6					1.5			
11472	31.3			8.5					
12312	33.4		3.9						
12314†	38.7								1.5
13313	52.2	1.3							
13327	39.6	32.9							
13332†	45.8								37.1
13334	51.1				25.6				
13369	48.4		1.1						
13382†	52.6								7.0
13383	48.9	8.7		13.5					
13411	62.8			6.4					
13586	38.1			33.5					
13587†	35.2					41.9		10.1	
14335	49.9			11.2					
14656†	32.1					19.2			
14657	34.0	26.4		22.2					
15665	49.9		8.6						
Total		100	100	100	100	100	100	100	100
*n*χ^2^		NA	NA	1.745	1.755	1.576	1.628	1.531	1.610

† Flanking frames not originally selected by a method.

## Data Availability

There is no new experimental data associated with this paper. The *AF* structure used (AF-Q06187) is available directly from the *AlphaFold* website (https://alphafold.ebi.ac.uk). The SASBDB entry (SASDF83) is available directly from the SASBDB website (https://www.sasbdb.org). All files generated by the website can be freely downloaded. The files generated in the website testing described here can be made available upon request to EB. The *SAXS-A-FOLD* software is available in a GitHub repository (https://github.com/ehb54/saxsafold).
