# Supplementary material for: SAXS-A-FOLD: a website for fast ensemble modeling optimizing the fit of AlphaFold or user-supplied protein structures with flexible regions to SAXS data
Source: J Appl Crystallogr. 2025 May 29;58(Pt 3):1034–49. doi: 10.1107/S1600576725003590 (PMC12135990; doi:10.1107/S1600576725003590)
Supplement: Supplementary file 1 [file j-58-01034-sup1.pdf]

JOURNAL OF
APPLIED
CRYSTALLOGRAPHY

Volume 58 (2025)

Supporting information for article:

SAXS-A-FOLD: a website for fast ensemble modeling optimizing the fit of *AlphaFold* or user-supplied protein structures with flexible regions to SAXS data

Emre Brookes, Joseph E. Curtis, Aaron Householder and Mattia Rocco

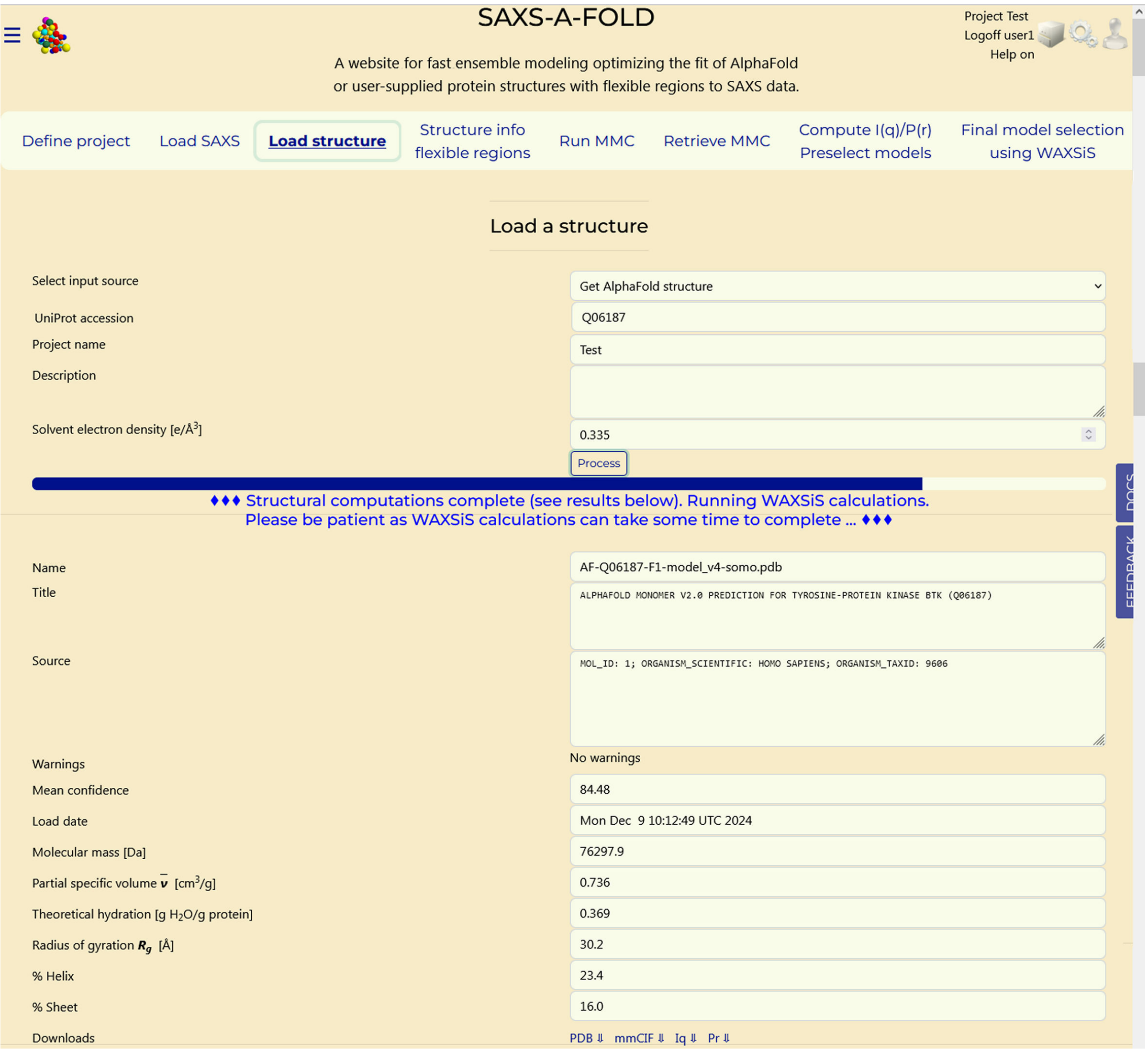

Figure S1 The ‘Load Structure’ Tab, showing the progress after uploading the Q06187 AF2 structure directly from their website. Shown is the output of the US-SOMO processing of the structure, while the progress bar monitors the WAXSiS computation of the $I(q)$. The final graphical outputs are shown in Fig. 4 in the main text.

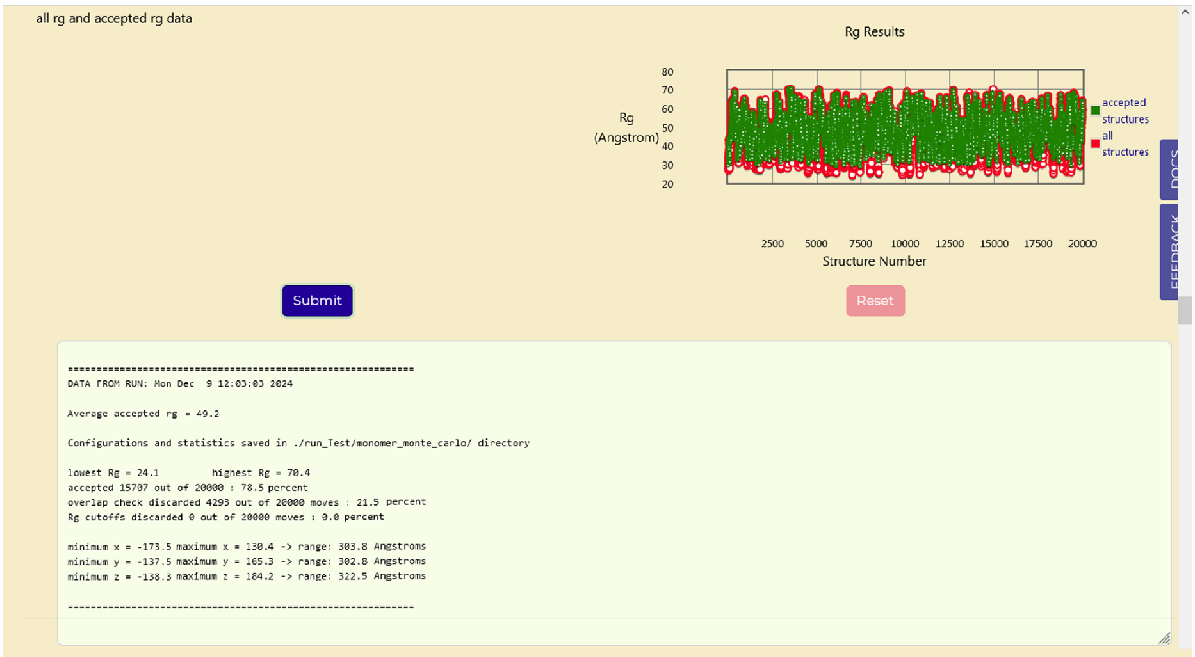

Figure S2 Graphical and numerical report of a MMC run provided at the bottom of the 'Run MMC' Tab upon completion. The interactive graph reports the distribution of the R_g of the generated models as a function of frame number (green squares, accepted models, red squares, all models). The text panel provides the statistics of the run.

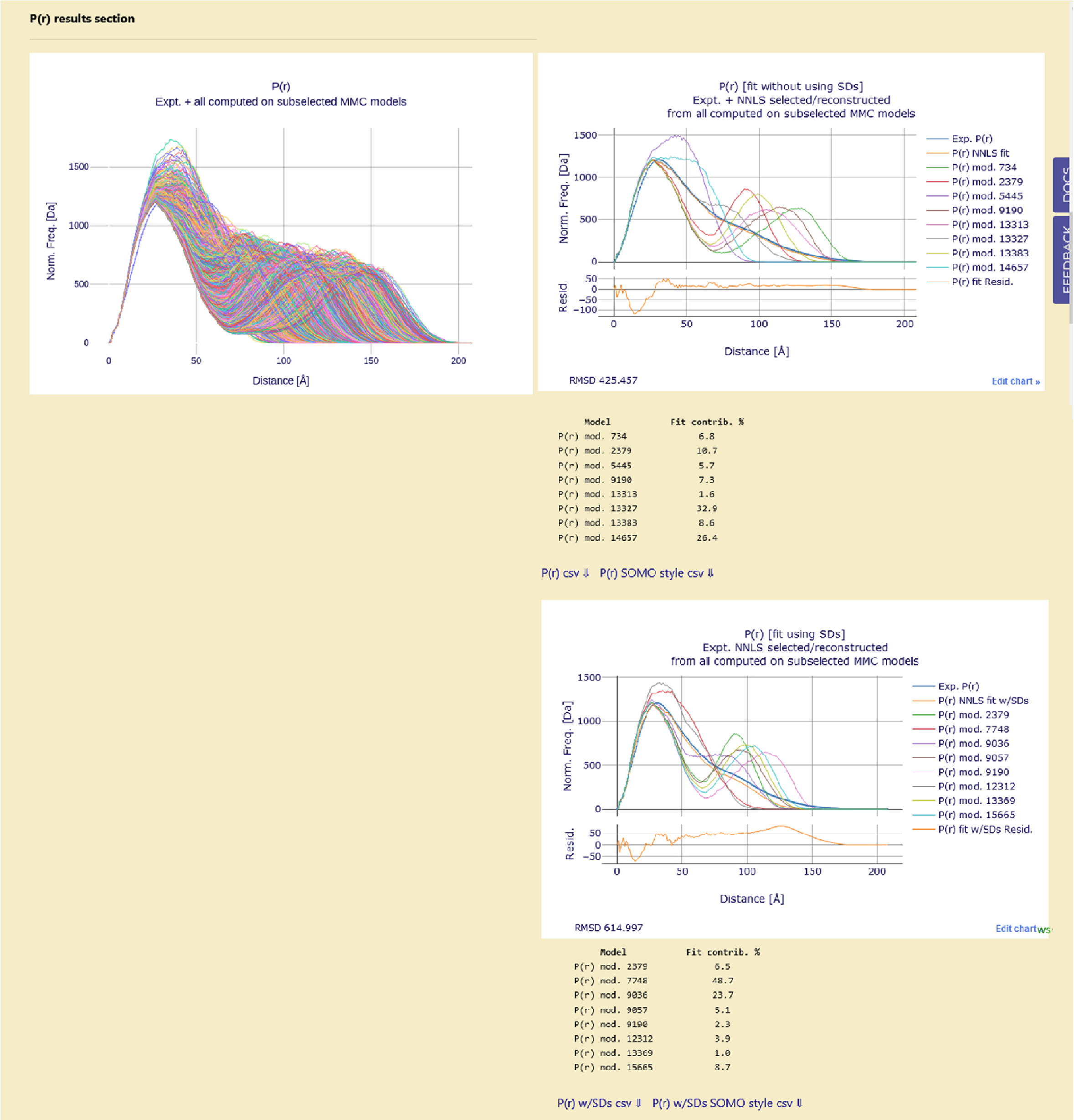

Figure S3 Results of the ‘Compute I(q)/P(r), preselect models’ Tab, part 1. Top-left, an image of the results of the $P(r)$ calculations for all the preselected models. Top right graph, the NNLS selection of the contributing models, without weighting by the SDs associated with the experimentally-derived curve. The experimentally-derived curve is blue, the contributing models, listed below it with their percent contribution, are in various colors, with the NNLS fit in orange (associated residuals plotted below it). Bottom-right graph, the NNLS selection of the contributing models, with weighting by the SDs associated with the experimentally-derived curve; below it, the list of the models and their percent contribution (same color coding as in the graph above). All the calculated $P(r)$, and the resulting NNLS fits, can be downloaded in two formats (in columns, or in lines, the latter being the format recognized by US‑SOMO) from the links provided below the models selected lists.

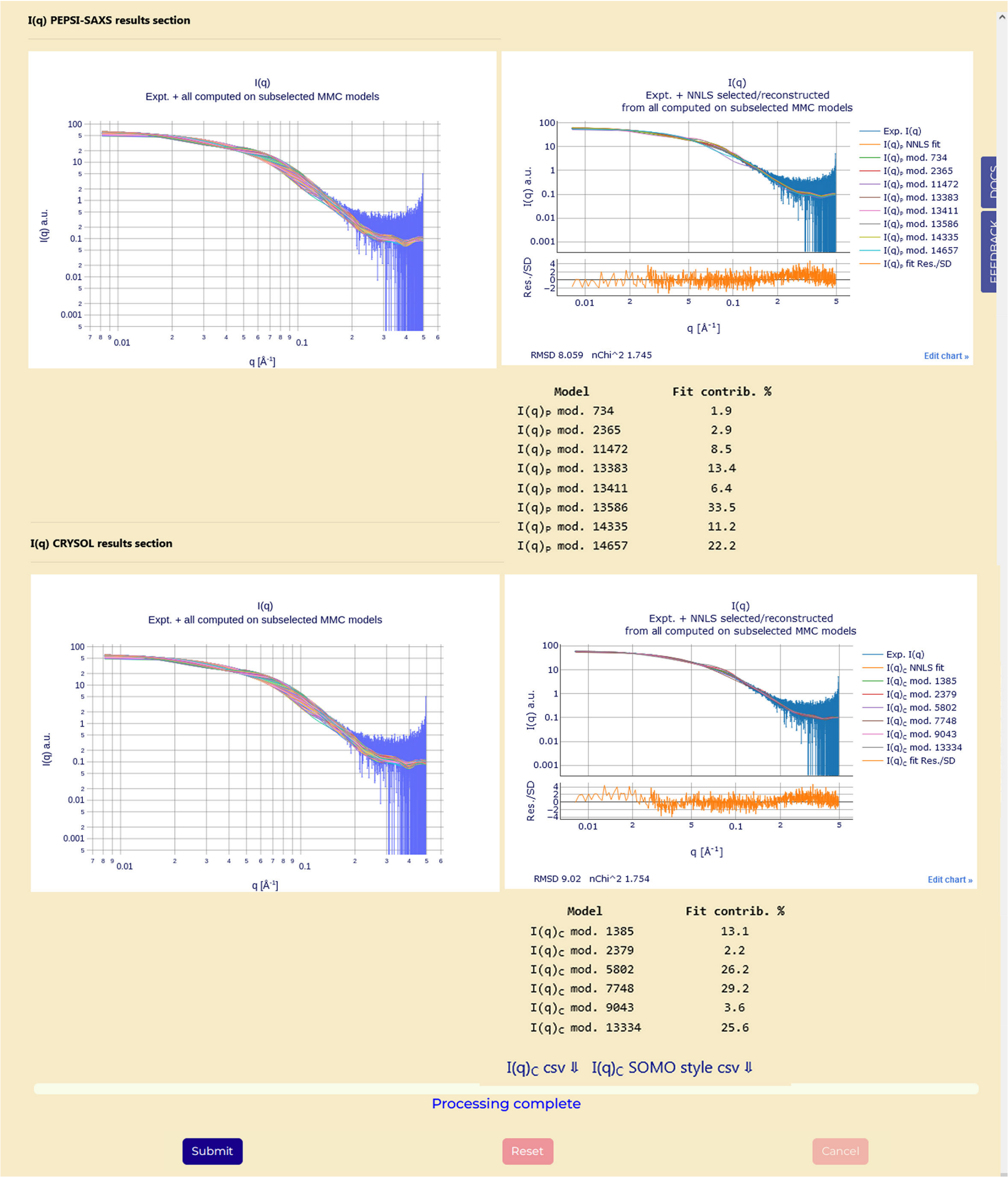

Figure S4 Results of the 'Compute I(q)/P(r), preselect models' Tab, part 2. Top-left, an image of the results of the *I*(*q*) calculations for all the preselected models using PEPSI-SAXS. Top-right graph, the NNLS selection of the contributing models. The experimentally-derived curve is blue, the contributing models, listed below it with their percent contribution, are in various colors, with the NNLS fit in orange (associated residuals/SD plotted below it). Bottom-left, an image of the results of the *I*(*q*) calculations for all the preselected models using CRYSOL. Bottom-right graph, the NNLS selection of the contributing models; below it, the list of the models and their percent contribution (same color coding as in the graph above). All the calculated *I*(*q*), and the resulting NNLS fits, can be downloaded in two formats (in columns, or in lines, the latter being the format recognized by US-SOMO) from the links provided below the models selected lists. The progress bar at the end will monitor the advancement of the calculations during processing.

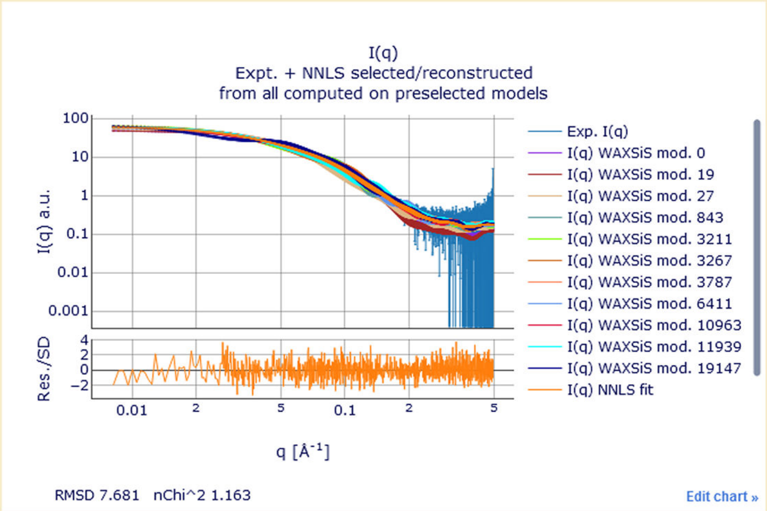

Model	Fit contrib. %
I(q) WAXSiS mod. 0	1.4
I(q) WAXSiS mod. 19	7.6
I(q) WAXSiS mod. 27	10.3
I(q) WAXSiS mod. 843	9.4
I(q) WAXSiS mod. 3211	9.0
I(q) WAXSiS mod. 3267	0.7
I(q) WAXSiS mod. 3787	34.8
I(q) WAXSiS mod. 6411	7.0
I(q) WAXSiS mod. 10963	14.8
I(q) WAXSiS mod. 11939	2.9
I(q) WAXSiS mod. 19147	2.0

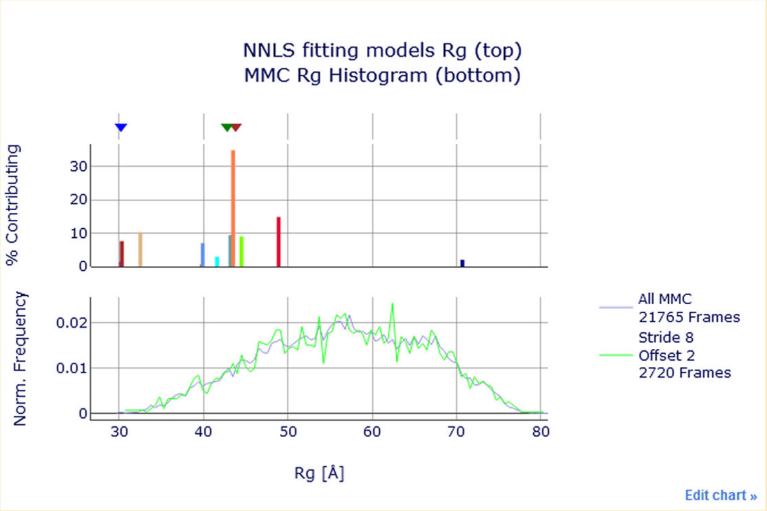

Figure S5 The WAXSiS-based final model selection on the Q06187/SASDF83 system with two flexible regions, residues 82-93 and 170-214. Top graph, the NNLS fit with all contributing models, listed with their percent contribution below the graph (various colors), and the resulting fit (orange) with the residuals/SD plotted below. Bottom graph, in the first plot the percent contribution vs. R_g values for the selected models (bars, hovering the mouse will reveal model number, percentage, and R_g) and three inverted triangles reporting the R_g values of the starting structure (blue), the weighted average of the selected models (green), and that calculated from the experimentally-derived $P(r)$ (brown). In the second plot, the R_g values frequency of the entire pool and of the sub-selected pool are reproduced.
